# Measurement invariance of Attention Deficit/Hyperactivity Disorder symptom criteria as rated by parents and teachers in children and adolescents: A systematic review

**DOI:** 10.1371/journal.pone.0293677

**Published:** 2024-02-23

**Authors:** Alexandra Garcia-Rosales, Samuele Cortese, Silia Vitoratou

**Affiliations:** 1 MRC Social Genetic Developmental and Psychiatry Centre, King’s College London, Institute of Psychiatry, Psychology, and Neurosciences, London, United Kingdom; 2 Psychometrics and Measurement Lab, Department of Biostatistics and Health Informatics, Institute of Psychiatry, Psychology, and Neurosciences, King’s College, London, United Kingdom; 3 Universidad Autónoma de Madrid, Madrid, Spain; 4 Kensington & Chelsea Child and Adolescent Mental Health Service, Central and North West London NHS Foundation Trust, London, United Kingdom; 5 School of Psychology, Centre for Innovation in Mental Health (CIMH), Faculty of Environmental and Life Sciences, University of Southampton, Southampton, United Kingdom; 6 Hassenfeld Children’s Hospital at NYU Langone, New York University Child Study Center, New York, New York, United States of America; 7 Division of Psychiatry and Applied Psychology, School of Medicine, University of Nottingham, Nottingham, United Kingdom; 8 Horizon Centre, CAMHS West, Solent NHS Trust, Southampton, United Kingdom; City University of New York, UNITED STATES

## Abstract

This systematic review aimed to establish the extent to which each Attention Deficit/Hyperactivity Disorder (ADHD) symptom criterion is being assessed without being influenced (biased) by factors such as informant, sex/gender, and age. Measurement invariance (MI) testing using confirmatory factor analysis (CFA) is the prime statistical method to ascertain how these factors may affect the measurement and colour the perception or interpretation of symptom criteria. Such effects (non-invariance) can be operationalised in the form of altered association of a symptom criterion with the measured trait (expressed via variations in CFA loadings which represent the weight of each symptom criterion) due to the factor(s) and/or artificially alter the probability of endorsement of a particular symptom criterion (expressed via variations in the CFA threshold(s) representing how mild or severe a given symptom is). Based on a pre-registered protocol (CRD42022276105), we searched PubMed, Global Health, Embase and PsycInfo up to 21-02-23 for studies that included MI assessments on specific ADHD symptom criteria in individuals aged 0–18 years old, using parental and/or teacher report. Self-reports were excluded, given the poor reliability of self-report in ADHD. All included studies met specific COnsensus-based Standards for the selection of health Measurement Instruments (COSMIN) criteria. Results were synthesised in tabular form, grouping results by factors (e.g. informant) from 44 studies retained. Most comparisons indicated both metric (same loadings) and scalar invariance (same thresholds) with regard to informant, gender, age, temporal (repeated assessments) and co-morbidity. Therefore, the available evidence supports the current diagnostic criteria. However, findings could have been improved by systematic reporting of the direction of bias and its effect size. There appears to be a bias towards reporting MI instead of non-invariance. More studies in the literature are needed where the amalgamation of information provided by different informs and the association of specific symptoms with comorbidity are analysed.

## 1. Introduction

ADHD is one of the most frequently diagnosed child and adolescent psychiatric disorders in clinical practice, affecting about 5 to 7% of school-aged children [1]. The 18 diagnostic items for ADHD were selected by the Diagnostic and Statistical Manual (DSM-IV) revision committee [2] based on the results of published articles and the field trials conducted specifically for the revision process. Extensive clinical experience and research evidence validate their use. The 18 criteria have generated reliable and consistent prevalence estimates across different geographic settings [3]. Feedback from clinicians indicated that the criteria might be further refined for routine clinical use [4]. Considerations concerning the utility of the DSM-IV, as well as the subsequent DSM-V criteria, were subsequently increasingly reported in the literature.

First, both DSM-IV [2] and DSM-5-TR) [5] classifications assume equal weighting of the 18 symptom criteria, nine relating to Inattentiveness (IA) and nine relating to Hyperactivity/Impulsivity (HI) with a diagnostic threshold set at the additive sum of symptoms present.

Second, the diagnostic manuals and any practice guidelines recommend collecting information for diagnostic purposes across informants and settings, specifically from the home and school settings, with information typically derived from parents and teachers [6]. However, the agreement between parent and teacher ratings of ADHD symptoms is generally low to moderate [7]. Tripp et al. [8] found that parent ratings were similar between children regardless of whether they were diagnosed with ADHD, but teachers outperformed parents regarding diagnostic discrimination. Hartman et al. [9] reported that teachers present less bias in ADHD ratings. This concurs with the publications by Vitoratou & Garcia-Rosales et al. [10] and Garcia-Rosales & Vitoratou et al. (2021) [11]. In these publications, the authors conclude that parents and teachers fundamentally observe different behaviours. In other words, the behaviours observed are situation-specific, i.e., home versus school.

Third, ADHD diagnostic assessments are further complicated by sex differences in ADHD presentation. Please note that from this point on, we will be using gender and sex indistinctly, as most of the literature reviewed predates the current awareness of sex and gender bias and the distinction between the sex assigned at birth and the gender one identifies with. In their meta-analysis on gender differences in ADHD, Gaub and Carlson [12] highlighted the need for further research looking at sex differences, minimising any potential source of bias (i.e. referral bias). According to Rucklidge’s view [13], there should be further research to develop “gender-appropriate diagnostic criteria” and “diagnostic tools”. DSM-IV [2] and DSM-5 [5] use symptom criteria cut-offs regardless of gender, whereas Conners questionnaires [14], commonly used to estimate ADHD symptoms severity, are standardised differently for boys and girls as well as according to age. Mick et al. [15] and Biederman et al. [16], describe an age-dependent symptom decline more pronounced for hyperactivity-impulsivity as subjects grow older. This natural evolution has recently been considered by DSM-5-TR [5], only requiring five symptom criteria to be present for each symptom dimension (IA or HI) to meet the diagnostic threshold. The detected differences across demographic groups in the total number of symptoms (score differences) have prompted some authors to propose adjusting the criteria threshold according to age [17] and gender [13, 18–20]. Rucklidge [13] emphasises the different patterns of comorbidity and impairment in the different genders, with girls displaying more internalising disorders (for example, anxiety, depression) and boys more externalising disorders (for instance, oppositional-defiant disorder, conduct disorder). However, some symptoms may be more discriminating or indexing greater severity in latent ADHD symptom dimensions [10, 21].

Fourth, co-occurring disorders are the norm in ADHD. Takeda et al. [21] have also explored the association of other factors, such as child socioeconomic status, academic impairment and co-occurring disorders, which might account for this discrepancy. Garcia-Rosales et al. [22] have identified 4 ADHD symptom criteria associated with Conduct Disorder (CD) comorbidity, for example.

To date, no established guidance exists to inform clinicians and researchers whether the presence of factors such as age, gender, informant, and co-occurring diagnoses affect the odds of endorsing a specific symptom criterion and to what extent. There is, however, evidence in the literature that such bias is to be expected, as referenced above.

In summary, the effects of age, sex, informant assessment and co-occurring conditions on the endorsement of ADHD symptom criteria have been evaluated in a substantial body of studies. Most of the research examined the extent to which the total number of symptoms can vary according to these factors, with the ensuing effects on diagnostic prevalence. It is, therefore, paramount to establish whether the information on the different ADHD criteria is biased or not by informant, age, gender, and co-occurring disorders. The symptom criteria (for instance, C*areless)* stems from the underlying trait (IA or HI), which fundamentally cannot be measured objectively, as we would a tumour in a pathology sample. We use proxies in scales reliant on informants, which help us ascertain whether a symptom criterion is present or absent. The question is whether these scales are reliable and what factors may affect their reliability and validity, such as informant, gender, age, and co-occurring disorders.

Measurement invariance (MI) assessments are statistical methods that enable us to answer this question. MI refers to the “extent to which the content of each [survey] item is being perceived and interpreted in the same way across samples” [23, p156]. MI refers to fair, unbiased measurement of a latent trait. That is, for instance, the probability of endorsing a symptom criterion for a trait should only reflect the trait rather than being affected by group memberships of the individual, such as sex, ethnicity, and co-occurring diagnoses, to name a few potential bias-inducing factors. For example, if one were to test weight differences in boys versus girls, one would want to establish first that the weighting scale used is not affected by one’s sex. That would ensure fairness, unbiasedness, impartiality, or in other words, sex invariance in the measurement weight. Only then would one be able to compare the differences in weight due to sex. Measurement invariance is a property of the measurement tool and not of the trait.

Confirmatory factor analysis methods are commonly used to investigate potential measurement bias due to group membership, such as using multiple group CFA model, or the multiple indicators multiple causes model (MIMIC) [24, 25] In the Item-Response Theory (IRT) context, the term used more often is differential item functioning (uniform or non-uniform DIF) and there is overlap within the two methods. Other CFA-based methods have been suggested in the literature, summarised in Somaraju et al. [26]. Leitgöb et al. [27] also discuss in detail recently suggested methods outside the CFA framework, such as approximate measurement invariance methods or methods utilising multilevel data models, which are useful in the presence of large number of groups. In this work, we focus on CFA based models (for categorical data) and IRT, which occur in the ADHD literature up to this point in time.

For an example of measurement invariance evaluations of the ADHD symptom criteria in a CFA framework, we refer the reader to the work of Vitoratou & Garcia-Rosales et al., 2019 [10], summarised in Table 1, where the factor model parameters are interpreted, and the four successive levels of measurement invariance are explained in detail. In summary, first, the model that fits the data best needs to be the same across groups (say sex or ethnicity groups, for example) or conditions (multiple raters or multiple assessments, for instance), and this is referred to as ‘configural’ invariance. Once configural invariance is established, the next level is the ‘metric’ (or ‘weak’ or ‘loadings’) invariance, which refers to how strongly each symptom criterion (item) is related to the underlying trait. Once metric invariance is established, the next step is exploring whether the probability of endorsing a symptom criterion is the same regardless of group membership or condition (‘scalar’ or ‘strong’ or ‘thresholds’ invariance). Finally, once the three first levels are established, the following step is exploring ‘strict’ invariance (‘residuals’ invariance), which refers to the amount of the variability of a symptom criterion that is left unexplained by the model and is typically not assessed for categorical data. The four types of measurement invariance can be assessed using the multiple group CFA model. Within the IRT context, the DIF techniques (uniform and non-uniform) correspond to the first three levels. The MIMIC approach often used in the literature also accommodates more than one external factor (often referred to as exogenous variables or covariates, adjusting for each other). MIMIC can be used with continuous variables (for example, age in years) rather than groups. It is of note that the MIMIC model takes both the configural and metric invariance for granted and explores the scalar invariance directly.

**Table 1:** Abbreviations of DSM-IV/5 item.

Inattention		Hyperactivity	
*Careless*	“Often fails to give close attention to details or makes careless mistakes in schoolwork, at work, or with other activities”	*Fidgets*	“Often fidgets with or taps hands or feet, or squirms in seat”
*Attention*	“Often has trouble holding attention on tasks or play activities”	*Seat*	“Often leaves seat in situations when remaining seated is expected”
*Listen*	“Often does not seem to listen when spoken to directly”	*Runs/Climbs*	“Often runs about or climbs in situations where it is not appropriate (adolescents or adults may be limited to feeling restless)”
*Instructions*	“Often does not follow through on instructions and fails to finish schoolwork, chores, or duties in the workplace (e.g., loses focus, side-tracked)”	*Motor*	“Is often "on the go" acting as if "driven by a motor‴
*Disorganised*	“Often has trouble organizing tasks and activities”	*Quiet*	“Often unable to play or take part in leisure activities quietly”
*Unmotivated*	“Often avoids, dislikes, or is reluctant to do tasks that require mental effort over a long period of time (such as schoolwork or homework)”	*Talks*	“Often talks excessively”
*Loses*	“Often loses things necessary for tasks and activities (e.g., school materials, pencils, books, tools, wallets, keys, paperwork, eyeglasses, mobile telephones)	*Wait*	“Often has trouble waiting his/her turn”
*Distracted*	“Is often easily distracted”	*Blurts*	“Often blurts out an answer before a question has been completed”
*Forgetful*).	“Is often forgetful in daily activities”	*Interrupts*	“Often interrupts or intrudes on others (e.g., butts into conversations or games)”

Whenever a symptom criterion is non-invariant (that is, different loadings and/or different thresholds), it is helpful to clarify the direction of the bias, for example, if the loading of the symptom *careless f*or girls is larger than the one for boys, or whether boys have a lower threshold (less odds) for endorsing a given symptom criterion, even though we assume the same levels of the trait for both genders. However, it is also important to report the size of the effect as large samples can produce statistically significant yet not clinically important differences [28]. The past few years several methods and coefficients have been proposed in the literature to quantify the effect size of non-invariant parameters see for instance Nye & Drasgow, (2011) [28]; Nye et al., (2019) [29]; Gunn et al, (2020) [30]). To be able to compare the scores between members of different groups (for instance, boys versus girls), it is important first to establish the measurement invariance of the criteria used in a similar way that one would need to establish the fairness of a weighting scale before comparing the weights of groups of people, in our previous example. On the other hand, the study of potential measurement non-invariance also enables us to understand differences across groups in their contributions to the trait of the symptom criteria and ascertain the direction of the bias for a given symptom criterion. This refined understanding of the criteria could directly inform the diagnostic process and potentially shift our focus on specific criteria as part of our assessment depending on informant, gender, age, and comorbidity of a given patient if the findings were to be generalised. It would also enable us to disregard potential items that might be redundant depending on the informant, gender, age and comorbidity.

Therefore, measurement invariance studies in ADHD need to be reviewed to ascertain convergent and divergent findings to inform any revisions of the diagnostic criteria for ADHD and day-to-day clinical practice. Such knowledge can then support clinical day-to-day diagnosis by looking at the differential information provided by the symptom criteria according to age, gender, informant, and comorbidity.

Testing for measurement invariance plays a paramount role in nosographic research, ensuring that comparisons across various groups of participants are both meaningful and valid.

The overarching aim of this systematic review was to identify symptom criteria that are consistently reported as measurement non-invariant for a given group membership or condition, using latent variable models methodology. Particularly, we aimed to identify the number of times each symptom criterion was reported in the available literature to be biased, depending on the informant (parent, teacher, mother, father), age (for instance, children versus adolescents), sex/gender, and co-occurring disorders (for example conduct disorder, anxiety).

## 2. Methods

The protocol for this systematic review PROSPERO 2022 CRD42022276105 was registered on Prospero. https://www.crd.york.ac.uk/prospero/display_record.php?RecordID=276105 PRISMA guidelines were followed; please see the supplemental documents with the PRISMA checklist for full details.

### 2.1 Eligibility criteria

We included studies that used factor analysis and/or differential item functioning to assess measurement invariance in ADHD. These procedures are part of the latent variable model methodologies. The inclusion and exclusion criteria were as follows:

#### 2.1.1 Inclusion criteria

Only papers published in scientific journals and dissertations in English, French or Spanish (due to lack of funding to translate papers in other languages) were included. The studies used latent variable models to assess the measurement invariance of the 18 symptom criteria of samples of children and young people between 0 and 18. This age bracket was chosen in the context of significant changes in the developing brain in childhood and adolescence. Measurement variance or non-invariance was determined with respect to age, sex or gender, informant (parent and/or teacher information were only considered) and co-occurring psychiatric diagnoses. Co-occurring psychiatric diagnoses were only considered where a binary choice was made: presence or absence of a disorder and/or clinical diagnosis.

We considered studies that employed the two independent factors models for ADHD (IA and HI considered as separate dimensions, constituted by nine symptom criteria each) based on DSM-IV (2) or with any symptom criteria of ADHD pertaining to DSM-III/IV criteria. Please note that we will refer to the DSM-IV/5 [2, 5] diagnostic criteria for ADHD. The abbreviations of DSM items used in this report are adapted from those used in the DSM-IV field trial [31] and are listed in *italics* in Table 1 below.

#### 2.1.2 Exclusion criteria

We excluded studies in adults and studies relying on self-report measures. Many children and adolescents with ADHD tend to under-report their symptoms and minimise their difficulties [32, 33]. Studies which did not use latent variable modelling in the assessment of measurement invariance of individual ADHD symptom criteria as described in DSM. In addition, studies with non-humans and treatment response studies (as opposed to diagnosis/understanding of ADHD symptom criteria articles) were filtered out. Furthermore, we did not include conference abstracts or book chapters.

### 2.2 Search methods for identification of studies

We initially searched on 24-10-21 Medline, PsycInfo, Embase and Global Health (to include grey literature). Search terms focused on ADHD, Symptoms, Home/Parents, School/Teachers and Item Factor Analysis (IFA). Please see details of the search in supplementary materials (S1 File). We chose to use, and in the search algorithm ‘and’ as literature is abundant on factor analysis in ADHD, primarily focusing on symptom dimensions, i.e., inattentiveness, hyperactivity and impulsivity. However, symptom dimensions were not the object of this measurement invariance study; our focus was on specific individual DSM criteria and how the appraisal of these could be altered depending on informants, settings and other co-variates. The search was subsequently updated. The searches were periodically updated, until on 21-02-2023. The Endnote software was used to pool the references list and filter out repeated references.

### 2.3 Screening/extraction, study quality assessment and reporting

AGR, Eric Taylor screened and extracted studies independently in the early stages of the project and SC subsequently. Disagreements were solved via discussion/ arbitration by SC. Study authors were contacted to clarify any doubt/request missing information.

All the studies met the following COSMIN [34] quality criteria relevant to this piece of work in terms of study design, the research aim, the construct to be measured, the target population for which the measurement instrument was developed, as well as the origin of the construct were clearly described. There was a clear description of the structure and scoring of the measurement instrument, a clear description of the evidence of the quality of the measurement tool and its context of use. The target population was clearly described in terms of inclusion and exclusion criteria to select raters, the methods used to choose them, and the study sample represented the target population. Regarding structural validity, confirmatory factor analysis was performed consistently in the methodology to carry this out, specifying the criteria for model fit. More importantly, the measurement invariance criteria as detailed in COSMIN needed to be fulfilled into the category of ‘very good’ according to COSMIN, with regards to a clear description of the group variable, including dichotomisation or categorisation, a clear description of the relevant characteristics of the patients that should be similar in both sub-groups, such as demographic or disease characteristics and the analyses were carried out with an appropriate number of patients.

The following data were extracted for each comparison (for instance. invariance in relation to age in the teacher ratings in a given article): article reference, demographics of the sample, psychometric tools used, type of informants, type of comparison (for example, invariance due to age in parents), the latent variable method used (multiple group confirmatory factor analysis, MIMIC or DIF), the type of model used (unidimensional, bifactor, 2-factor analysis or 3 or more factors). The level of measurement invariance (configural, metric, scalar) established for each symptom criterion was specified. If there was non-invariance, the direction of bias was specified (for instance, lower threshold boys rather than girls for a given symptom). No assumptions were made when there was missing information. All the comparisons were logged into an access database, which was subsequently exported to Excel, and then to SPSS for data analysis.

Our data is available on the OSF data repository as follows: DOI 10.17605/OSF.IO/E8VTZ

Subsequently, the different types of comparisons were categorised (for example, invariance in relation to informant, age, and gender) to ascertain the number of comparisons across different studies and log these numbers in the review. The concept of a small or large number of comparisons can be subjective, so we have specified the number of comparisons throughout.

Unless otherwise specified, most studies used dependent samples where the same group of children are, for example, assessed by two different informants (for instance: parents and teachers). When the samples were independent, both informants assessed two different samples, i.e., two different groups of children.

## 3. Results

**Fig 1:**
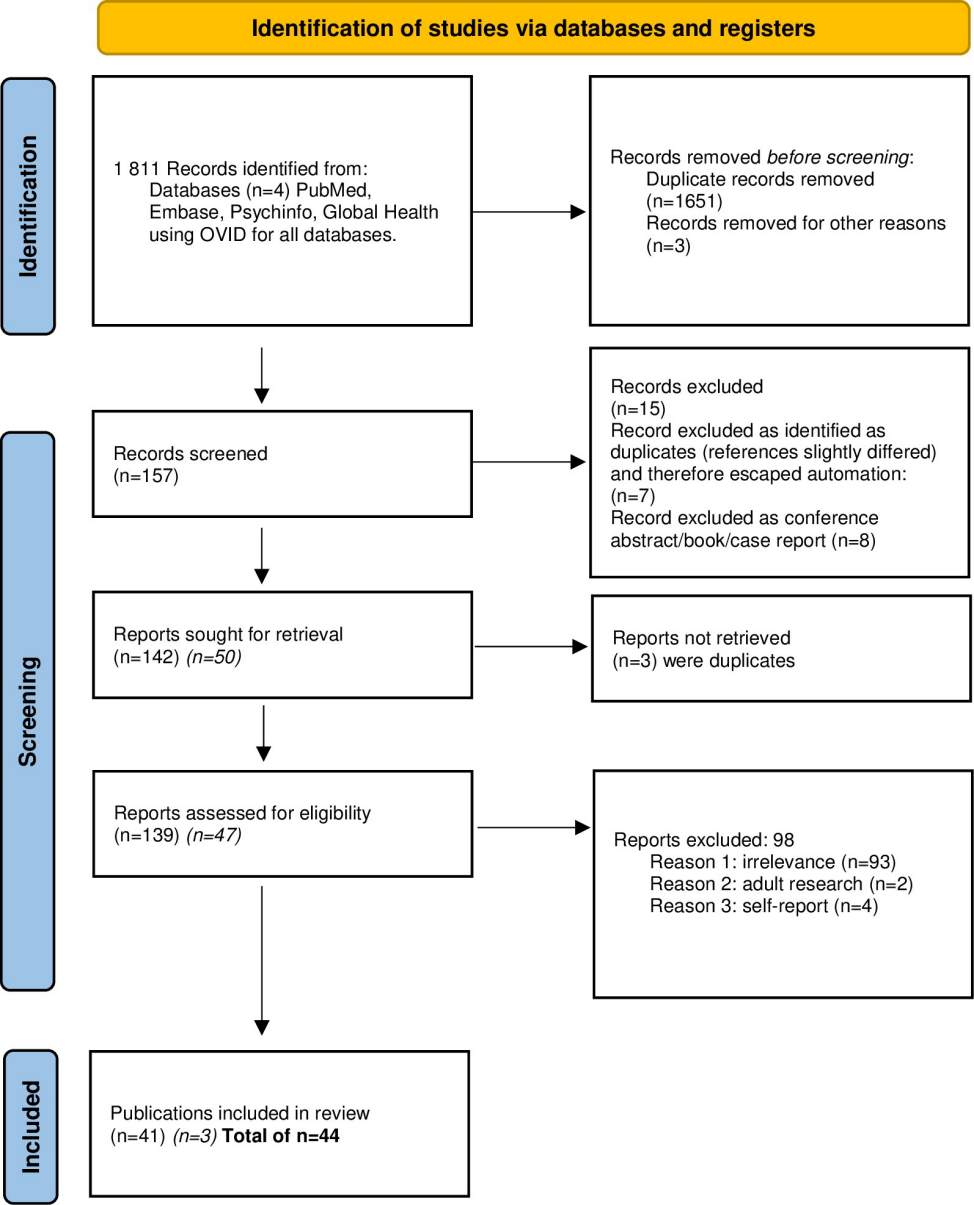
Prisma flowchart. Note: *Italics*: *second search*. Please note that in the second search, the vast majority of duplicates/excluded articles were the same as in the first search or were articles that had been included in the first search. Only 4 new articles were excluded, and they are referenced in S1 Table.

As shown in the PRISMA flowchart (Fig 1), from an initial 157 potentially relevant references, we retained 41 unique studies. When the second search was conducted, AGR and SC independently screened 47 articles and included three new studies, with 100% agreement between AGR and SC. Please see S2 File and S1 and S2 Tables for the included and excluded studies lists and a table detailing the included publications’ characteristics.

All the included studies met the COSMIN criteria specified in the previous section. Some studies only focused on IA as opposed to HI; some included only DSM-III [35] criteria, and others included symptom criteria in their exploratory factor analysis, which may have sometimes included only some DSM-IV criteria. In addition, the direction of the bias was not systematically reported in terms of loadings and thresholds.

As part of drafting this paper, the authors used tables that included the list of publications in any given invariance category, the psychometric tool used, the type of model used, and the type of sample (community versus clinical). A different table was drawn to synthesise information for each symptom criterion regarding measurement invariance, for example, according to age or according to gender. The number of comparisons is logged in terms of metric and scalar invariance, and when there was non-invariance, the direction of bias was specified. This enables us to draw overarching conclusions for any given symptom criterion. For example, *careless* was identified as gender invariant regarding equality of loadings in all the comparisons, which means that this symptom criterion carries the same weight in boys and girls. The next step would be to identify whether boys and girls have the same threshold for endorsing the symptom *careless*. If thresholds are equal, scalar invariance can be established. If not, the direction of bias would need to be clarified regarding differences in thresholds between boys and girls.

To simplify the presentation of the results and avoid using multiple tables when there was a small number of comparisons, the authors presented this in narrative form instead of overwhelming the reader with numerous tables. All tables are elaborated based on the available and reported data. When the number of comparisons was large, we chose to design a table that would include the reference for each study, the model type, the number of factors that were in the model used, the psychometric tool used to assess ADHD symptoms, and the number of comparisons in the given publication.

### 3.1 Invariance in relation to informant

Table 2 presents the list of publications included in this review for assessing measurement invariance in relation to informant.

**Table 2:** Summary table of the informant invariance publications with the number of comparisons for each (total of 36 comparisons) and a specific focus on mothers versus fathers (10) as well as parents versus teachers.

Publication	Sample	Model	Scale	Number of comparisons	Mothers vs Fathers (dependent samples)	Parents vs Teachers
[36]	Community sample	2 factor model with ADHD-IA and Sluggish Cognitive Tempo	Child and Adolescent Behaviour Inventory (CADBI) parents and teachers	3	1	1
[37]	Community sample	5 factor model with IA, HI, ODD, academic competence and social competence	Child and Adolescent Behaviour Inventory (CADBI) parents and teachers	1	1	
[38]	Community sample	3 factor analysis with IA, HI and academic impairment	Child and Adolescent Behaviour Inventory (CADBI) parents and teachers	3	1	
[39]	Community sample	3 factor model with IA, HI and ODD factors	ADHD Rating Scale-IV (for ADHD symptom rating) and the ODD Section of the Disruptive Behavior Disorders Rating Scale (for ODD symptom rating)	6	2	
[40]	Clinical and non-clinical groups	Two factor model with IA and HI	ADHD-RS-IV parent and teacher versions	3		1
[41]	Community sample	Two factor model with IA and HI	ADHD Rating Scale-5 Home Version	6	1	
[42]	Community sample	5 factor model with ADHD-IN, ADHD-HI, ODB-Adult, ODB-Children and Academic Competence	Child and Adolescent Behaviour Inventory (CADBI) parents and teachers	1	1	
[43]	Community sample	Two factor model with IA and HI	Strengths and Weaknesses of ADHD symptoms and Normal Behavior Rating Scale (SWAN), used for parents and teachers.	8		8
[7]	Clinical sample	Two factor model with IA and HI	Vanderbilt ADHD Rating Scales for parents (VADPRS) and teachers (VADTRS)	1		1
[44]	Elementary school children	3 factor model ADHD-IN, ADHD-HI, ADHD-ODD	Child and Adolescent Behavior Inventory (CADBI) for parents	1	1	
[45]	School sample	3 factor model with cross-loadings including IA, HI and Impulsivity	Child and Adolescent Behavior Inventory (CADBI) for parents	2	2	
[10]	Clinical sample with their siblings	Two factor model with IA and HI	Hypescheme algorithm to ascertain symptom criteria as present or absent using Parental Account of Clinical Symptoms and the Conners Teacher	1		1

Our review included 36 individual comparisons and focussed specifically on mothers versus fathers and parents versus teachers. While there were a substantial number of studies reporting on measurement invariance in relation to mothers versus fathers:10 comparisons in Burns et al., 2017 [36]; Burns et al., 2009 [37]; Burns et al., 2014 [31]; Burns et al., 2013 [39]; de Moura et al [35], DuPaul et al., 2016 [41]; Preszler and Burns, 2019 [44]; Preszler et al., 2022 [45], Gomez, 2010 [46] in dependent samples and 8 in independent samples (Gomez, 2010 [46] and Khadka and Burns [47]), and parents versus teachers (12 comparisons), there were only a few studies for mother versus teacher (2 comparisons in Burns et al., 2013 [39]), father versus teacher (2 comparisons in Burns et al., 2013 [39]), multiple informants (1 comparison in Burns et al., 2014 [38]), maternal years of education (3 comparisons in Cogo-Moreira et al [48], primary school teachers versus secondary school teachers (1 comparison in Burns et al., 2017 [36]), teachers versus aides (1 comparison in Burns et al. 2014 [38]), mothers in independent samples (1 comparison in Khadka and Burns [47]) and fathers in independent samples (1 comparisons in Khadka and Burns, 2013 [47]), parental ethnicity (1 comparison in DuPaul et al., 2020 [49], parental cultural background (Trejo et al., 2022) teacher ethnicity (1 comparison in DuPaul et al., 2020 [49]), male teacher versus female teacher (2 comparisons in in DuPaul et al., 2019 [49]). Unless otherwise specified, all comparisons were made in independent samples, meaning that mothers and parents rated the same children instead of two separate groups of children. Given the small number of comparisons in most cases, it is not possible to draw overarching conclusions that would be useful for this review. We, therefore, focused below on the mothers versus fathers and parents versus teachers’ comparisons.

#### 3.1.1 Mothers versus fathers

There were ten comparisons for IA symptoms and 9 for HI, where both metric and scalar were established for all comparisons.. In one study (Burns et al., 2017) [36] in relation the assessment of measurement invariance was only reported only for Inattentiveness and not for Hyperactivity/Impulsivity.

In only two publications [46, 47], the mothers-versus-fathers comparisons were carried out using independent samples. There was a total of 8 comparisons (6 in Gomez, 2010 [46] and 2 in Khadka et al. [47]). Only 2 in Gomez (2010) [40] identified non-invariance for specific symptom criteria in relation to which parent was the informant (Table 1). In this publication, in one comparison using multiple group CFA and chi-squares, differences in loadings reported for specific items, fathers associated more strongly with the traits of three criteria (*attention*, *seats*, and *runs/climbs*) for the same levels of the traits. In contrast, mothers associated more strongly with the criteria *quiet* and *distracted*. In another MIMIC and chi-square comparison controlling for age and gender, *attention loses and runs/climbs* showed higher values for father ratings. In contrast, mother ratings were higher for *distracted*, *motor* and *talks*.

#### 3.1.2 Parents versus teachers

Overall, there was abundant evidence supporting metric invariance in all symptom criteria (please see Table 3). All exceptions were reported in Vitoratou & Garcia-Rosales et al. (2019) [10], the only study of ADHD cases with their siblings in this category.

a. In one comparison, the loadings for *careless*, *loses*, *forgetful*, *runs/climbs*, *quiet* and *blurts* were higher for parents than teachers. In contrast, *attention*, *wait*, and *interrupts* had higher loadings for teachers than for parents in one comparison.
b. Regarding scalar invariance, parents had a lower threshold for reporting *listens* and *distracted* (Vitoratou & Garcia-Rosales, 2019) [10], whereas teachers had a lower threshold for reporting *instructions*.
c. Scalar invariance was consistently established for *disorganised*, *unmotivated*, *fidgets*, *seats* and *talks*.

**Table 3:** Measurement (Non)-Invariance assessment: Parents (P) versus teachers (T). Where there is bias the direction of the bias is specified along the number of comparisons. Tables are elaborated based on the available and reported data.

Symptom criterion	Metric (weak) invariance			Scalar (strong) invariance		
	*Number of Comparisons*	*Invariant loadings*	*Direction of bias*	*Number of Comparisons*	*Invariant thresholds*	*Direction of bias*
**Inattentiveness**						
*Careless*	12	9	P>T: 1	4	4	
*Attention*	12	9	T>P: 1	4	4	
*Listens*	12	10		5	4	P>T: 1
*Instructions*	12	10		5	4	T>P: 1
*Disorganised*	12	10		6	6	
*Unmotivated*	12	10		6	6	
*Loses*	12	9	P>T: 1	5	5	
*Distracted*	12	10		6	5	P>T: 1
*Forgetful*	12	9	P>T: 1	5	5	
**Hyperactivity/Impulsivity**						
*Fidgets*	11	9		5	5	
*Seats*	11	9		5	5	
*Runs/Climbs*	11	8	P>T: 1	4	4	
*Quiet*	11	8	P>T: 1	4	4	
*Motor*	11	8	T>P: 1	5	4	
*Talks*	11	9		5	5	
*Blurts*	11	8	P>T: 1	4	4	
*Wait*	11	8	T>P: 1	4	4	
*Interrupts*	11	8	T>P: 1	4	4	

*In one study (Burns et al., 2017), only Inattentiveness was reported and not Hyperactivity/Impulsivity

### 3.2 Invariance in relation to sex/gender

Table 4 presents the list of publications included in this review concerning the assessment of measurement invariance in relation to sex/gender. There were 36 comparisons, 21 with parents as informants, 16 as teachers and two combining parents and teachers. Seven comparisons were carried out among the studies with DIF and 28 using Multiple Item Factor Analysis (MIFA).

**Table 4:** Summary table of the gender invariance publications (total of 22 tests) with the number of comparisons depending on informant (parents or teachers).

Publication	Sample	Model	Scale	Number of comparisons	Parents	Teachers
[50]	Community	bifactor model with a central dimension plus 3 specific factors (inattention, hyperactivity and impulsivity)	ADHD questionnaire (replicating 18 items of DSM-IV) in Teachers	1	0	1
[51]	Community	Factor analysis applied ot the 9 ADHD-IA symptoms	Child and Adolescent Behavior Inventory (CADBI) for parents	1	1	0
[52]	Community	A priori two-factor model on the 15 SCT symptoms and AHD-IA symptoms	Child and Adolescent Behavior Inventory (CADBI) for teachers	1	0	1
[53]	Community and Clinical	4-factor model; IA, HI, ODD and overt conduct disorder factors	Child and Adolescent Behavior Inventory (CADBI) for parents	1	1	0
[54]	Clinical	3-factor model with IA, HI and ODD	Child and Adolescent Disruptive Behavior Inventory (CADBI) parent version	2	2	0
[55]	Community	Bifactor model including one AHD G-factor and two specific S- factors (IA and HI)	ADHD Rating scale (ADHD-RS), teacher scale	1	0	1
[56]	Community	Two factor model with IA and HI, does not match fully with DSM-IV criteria with 6 IA items, 8 HI items and 2 combined	ADHD Symptoms Rating Scale (ADHD-SRS), parent version	1	1	0
[48]	High-risk and random sample	Bi-factor model including one general ADHD facto and 3 specific factors (Inattentiveness, Hyperactivity and Impulsivity)	DAWBA administered to biological mother by trained lay interviewer	1	1	?
[57]**	Twin register	1 factor solution with OPP (oppositional behavior), ATT (inattention/cognitive problems) and HYP (hyperactivity) and 2 factor solution for ADHD (Attention problems and Hyperactivity/impulsivity)	Conners’ Teacher Rating Scales- Revised (CTRS-R)	6	0	6
[58]***	Community	1 dimension of ADHD	Ontario Child Health Study Emotional Behavioural Scales: Teacher version (of children 4–13)	1	0	1
[40]	Clinical a non-clinical groups	Two factor model with IA and HI	ADHD-RS-IV parent and teacher versions	2	1	1
[49]	Community	Univariate for each subscale IA and HI.	ADHD Rating Scale-5 Home and School	2	1	1
[41]	Community	Two factor model with IA and HI	ADHD Rating Scale-5 Home Version	4	3	1
[59]	Community	Univariate for each subscale IA and HI.	DSM-IV ADHD rating Scale (DARS) parent scale	2	2	0
[60]	Community	Univariate for each subscale IA and HI.	Disruptive Behavior rating scale (DBRS) in parents	1	1	0
[61]	Community	3 correlated factors solution with IA, HI and ODD	Swanson, Nolan and Pelham scale version IV (SNAP-IV) for parents and teachers	1	1	1
[62]****	Clinical sample with diagnoses of ADHD or ASD	4-factor model with social communication factor, restricted and repetitive interests factor the hyperactivity impulsivity factor and the inattentive factor.	Swanson, Nolan and Pelham scale version IV (SNAP-IV) for parents	1	1	0
[63]	Twin pairs	3 factor model with IN, HI and Functional Impairment	Disruptive Behavior Rating Scale (DBRS) completed by parents	?	1	0
[64]	Community	Unidimensionality for each subscale IA, HI and ODD	ADHD-RS for parents and teachers	2	1	1
[65]	Clinical and community	Incomplete bifactor model	Fremdbeurteilungsbogen für Kinder and Jugendliche mit Aufmerksamkeitsdefizit-/Hyperaktivitätsstörung(FBB-ADHD)	1	1	0
[66]	Community	Bifactor model	Swanson, Nolan and Pelham scale version IV (SNAP-IV) for parents	1*	1	0
[10]	Clinical sample with their siblings	Two factor model with IA and HI	Hypescheme algorithm to ascertain symptom criteria as present or absent using Parental Account of Clinical Symptoms and the Conners Teacher	2	1	1

*Please note that comparisons were made using 6 different models where configural invariance was established, however only one model showed appropriateness of fit and measurement invariance.

** Only includes 12 ADHD criteria not all of which map onto DSM

*** Only includes 8 ADHD criteria that map onto DSM

**** only includes 16 ADHD DSM-IV items

#### 3.2.1 Invariance in relation to sex/gender, according to parents

Overall, there was metric invariance with respect to gender according to parents (please see Table 5). Measurement non-invariance with regards to gender was reported for *disorganised* (Başay et al. [51]), *loses* (Vitoratou & Garcia-Rosales et al. [10]), *talks* (2 comparisons in DuPaul et al., 2020 [49]) and Makransky et al. [64]) and *interrupts* (DuPaul et al., 2020 [49]), where females had lower thresholds than males and for *unmotivated* (Vitoratou & Garcia-Rosales et al. [10]), *fidgets* (DuPaul et al., 2020 [49]), *seats* (Gomez, 2012 [60]), *runs/climbs* (DuPaul et al., 2020 [49]), and *talks* (1 comparison in Vitoratou & Garcia-Rosales et al. [10]).

**Table 5:** Measurement (Non)-Invariance assessment: Males (M) versus Females (F) according to parents. Where there is bias, the direction of the bias is specified along the number of comparisons.

Symptom criterion	Metric (weak) invariance			Scalar (strong) invariance		
	*Number of Comparisons*	*Invariant loadings*	*Direction of bias*	*Number of Comparisons*	*Invariant thresholds*	*Direction of bias*
**Inattentiveness**						
*Careless*	20	19		19	19	
*Attention*	21	20		20	19	
*Listens*	19	18		18	18	
*Instructions*	21	20		20	19	
*Disorganised*	21	20		19	18	F>M: 1
*Unmotivated*	20	19		19	18	M>F: 1
*Loses*	21	20		19	18	F>M: 1
*Distracted*	21	20		20	19	
*Forgetful*	19	18		18	18	
**Hyperactivity/Impulsivity**						
*Fidgets*	20	19		18	17	M>F: 1
*Seats*	19	17		17	15	M>F: 2
*Runs/Climbs*	18	16		16	15	M>F: 1
*Quiet*	19	17		16	16	
*Motor*	18	17		17	17	
*Talks*	19	18		17	14	M>F: 1; F>M: 2
*Blurts*	18	17		17	17	
*Wait*	20	19		18	18	
*Interrupts*	18	17		17	15	F>M: 2

#### 3.2.2 Invariance in relation to sex/gender, according to teachers

There were equal loadings for all symptom criteria. Regarding IA, 8 out of 9 symptom criteria had equal thresholds. There was a lower threshold for girls compared to boys, according to parents, for *forgetful* (Vitoratou & Garcia-Rosales et al. [10]). Please see Table 6. With regards to IA, girls had lower thresholds than boys for *fidgets* (in two comparisons in DuPaul et al., 2016 [40] and Makransky et al. [64]) and *runs/climbs* (in one comparison in Makransky et al. [64]), whereas girls had a higher threshold for *talks* (in 3 comparisons reported in DuPaul et al., 2020 [49]; Makransky et al. [64] and Vitoratou & Garcia-Rosales et al. [10]). Therefore, there was invariance regarding gender in loadings and thresholds based on teachers’ report.

**Table 6:** Measurement (Non)-Invariance assessment: Males (M) versus Females (F) according to teachers. Where there is bias, the direction of the bias is specified along the number of comparisons.

Symptom criterion	Metric (weak) invariance			Scalar (strong) invariance		
	*Number of Comparisons*	*Invariant loadings*	*Direction of bias*	*Number of Comparisons*	*Invariant thresholds*	*Direction of bias*
**Inattentiveness**						
*Careless*	10	10		10	9	
*Attention*	16	16		16	15	
*Listens*	9	9		9	8	
*Instructions*	16	16		16	15	
*Disorganised*	15	15		15	14	
*Unmotivated*	15	15		15	14	
*Loses*	9	9		9	8	
*Distracted*	16	16		16	15	
*Forgetful*	15	15		15	13	F>M: 1
**Hyperactivity/Impulsivity**						
*Fidgets*	14	14		14	11	M>F: 2
*Seats*	14	14		14	13	
*Runs/Climbs*	14	14		14	12	M>F: 1
*Quiet*	8	8		8	7	
*Motor*	14	14		14	13	
*Talks*	8	8		7	4	F>M: 3
*Blurts*	8	8		8	7	
*Wait*	14	14		14	13	
*Interrupts*	14	14		14	13	

#### 3.2.3 Invariance in relation to sex/gender, according to teachers and parents combined

Parent and teacher information was combined in the study by Vitoratou & Garcia-Rosales et al. [10] (details of sample, model and psychometric tools specified previously). The authors used the ‘and’ and the ‘or’ rules described by Valo et al. [67]. Parents and teachers must agree that a given symptom criterion is present when using the’ and-rule’. When the ‘or’ rule is used, either parent or teacher scores the symptom criterion as present. When applying both rules, full metric invariance was established. When applying the ‘and-rule’ combining both parent teacher information, there was gender invariance in 17 of the 18 symptoms.

Regarding thresholds for the symptom talks, the endorsement was higher in girls than in boys. When using the ‘or-rule’, there was gender invariance in 15 out of 18 symptoms. Girls had a lower threshold for endorsement of *forgetful*, *loses* and *talks*.

### 3.3 Invariance in relation to age

There were a total of 24 comparisons, 13 where parents were informants, 8 with teachers and 3 with parents and teachers combined. The publications by Vitoratou & Garcia-Rosales et al. [10] and Narad et al. [7] are the only ones with comparisons where the information is combined (3 comparisons). Four comparisons were conducted with DIF, 15 with MIFA and 5 with MIMIC. See S4 Table for the summary table of age-related invariance publications (total of 20 comparisons) with the number of comparisons depending on informant (parents or teachers).

Please see S3 Table Summary table of the Age Invariance publications (total of 24 tests) with the number of comparisons depending on informant (parents or teachers).

#### 3.3.1 Invariance in relation to age, according to parents

For the parental reports, equal loadings were established for all symptom criteria, except for one comparison (Burns et al., 1997), in which *loses* where younger populations had a higher loading.

There was more disparity with to the thresholds. *Disorganised*, *distracted* and *forgetful*, *quiet*, *talks*, *wait*, and *interrupts* achieved scalar invariance consistently. Parents had a lower threshold *attention* in DuPaul et al., 2020 [49], *listens* in Makransky et al. [64], *instructions* in DuPaul et al., 2020 [49], *seats* in DuPaul et al., 2020) [49], and *runs/climbs* (in 2 comparisons reported in DuPaul et al., 2020 [49] and Vitoratou & Garcia-Rosales [10]). Parents reported a lower threshold for *unmotivated* in one comparison in younger children (DuPaul et al. 2020) [49] and in another in older children in Vitoratou & Garcia-Rosales et al. [10]. Parents had a lower threshold for reporting *careless* in Makransky et al. [64], *fidgets* [64], *motor* [10] and *blurts* [10] in older children and young people.

Please see S4 Table Measurement (Non)-Invariance assessment according to parents.: Younger (Y; less than 10 years old) versus Older (O; 11 years old and older) according to Parents.

#### 3.3.2 Invariance in relation to age, according to teachers

Regarding teacher ratings, equality of loadings was established for 100% of the comparisons.

Strong invariance was established for *attention*, *listens*, *disorganised*, *unmotivated*, *fidgets*, *seat*, *runs/climbs*, *quiet* and *motor*. In one comparison, teachers had a lower threshold for *loses* in Makransky et al. [64], *forgetful* in Vitoratou & Garcia-Rosales et al. [10], *talks* [10], *blurts* [10], *wait* [10] and *interrupts* [10] in older children. In 2 comparisons, younger children had a lower threshold for *distracted* in DuPaul et al., 2020 [49] and Makransky et al. [64] and in 1 comparison for *instructions* in DuPaul et al., 2020 [49], according to teachers.

Please see S5 Table Measurement (Non)-Invariance assessment: Younger (Y; less than ten years old) versus Older (O; 11 years old and older) according to Teachers.

### 3.4 Temporal invariance (repeated assessments)

We separated age from temporal invariance. Age invariance was established in cross-sectional studies comparing older versus younger children and young people. Temporal invariance was established in populations who underwent repeated assessments over time.

Please see S6 Table: Summary table of the Temporal (longitudinal) Invariance publications (total of 14 tests) with the number of comparisons depending on the informant (parents or teachers).

Given the number of comparisons, we will focus on temporal invariance according to parents (7 comparisons) on one hand and temporal invariance according to teachers (4 comparisons) on the other. Eleven comparisons were made using Longitudinal Item Factor Analysis (LIFA) and 3 using MIFA.

#### 3.4.1 Temporal invariance, according to parents

The included studies reported overall equality of loadings according to parental report and, in less than 50% of the comparisons, established equality of thresholds. However, the direction of the bias was not reported.

Please see S7 Table: Measurement (Non)-Invariance assessment: Repeated assessments according to Parents.

#### 3.4.2 Temporal invariance, according to teachers

In contrast to parental information, in 75% of the comparisons, scalar invariance was established based on teacher report for all symptom criteria.

Please see S8 Table: Measurement (Non)-Invariance in repeated assessments according to teachers.

### 3.5 Co-morbidity invariance

The publications by Cogo-Moreira et al. [42] and Vitoratou & Garcia Rosales et al. [10] are the only ones where co-morbidity invariance was examined. There were 14 comparisons, 12 of which are in the Vitoratou & Garcia-Rosales et al. [10] paper looking at Anxiety Disorder, Oppositional Defiant-Disorder and Conduct Disorder. It is the only paper where the ‘and’ and ‘or’ rules are used. Teacher ratings were not markedly biased in the presence of a co-occurring diagnosis. Parental ratings were more affected by co-morbidity, especially in the presence of ODD for HI items. When the information was combined, there were more measurement invariants when the ‘and- rule’ was used, as opposed to the ‘or-rule’.-

## 4. Discussion

We aimed to identify to what extent each ADHD symptom criterion was reported in the available literature to be biased, depending on the informant, sex/gender, and co-occurring disorders. Our study showed that equality of loadings and thresholds for all DSM-IV ADHD criteria was reported in most comparisons between mothers and fathers, primarily dependent samples, despite the heterogeneity of the models used: two-factor, three-factor, or five-factor models. There were some examples of measurement non-invariance when the samples were independent.

However, this was not the case between parents and teachers, with some comparisons indicating non-invariance. Scalar invariance was established between parents and teachers for *disorganised*, *unmotivated fidgets*, *seats*, and *talks*.

Regarding invariance related to gender, separately in parents’ and teachers’ reports, equality of loadings is reported in all cases but not for all thresholds. Scalar invariance in parents was established for *careless*, *attention*, *listens*, *instructions*, *distracted*, *forgetful*, *quiet*, *motor*, *blurts* and *waits* in parents. Scalar invariance in teachers was found for all symptom criteria apart from *forgetful*, *fidgets*, *runs/climbs* and *talks*.

Metric invariance was established regarding age separately for parents’ and teachers’ ratings. Scalar invariance for age according to parents’ ratings was established for *loses*, *distracted*, *forgetful*, *quiet*, *talks*, *wait* and *interrupts*. For teachers, scalar invariance was established for *attention*, *listens*, *disorganised*, *unmotivated*, *fidgets*, *seat*, *runs/climbs*, *quiet*, *motor* and *talks*.

Regarding repeated assessments, teachers appeared to be reliable informants achieving 100% of scalar invariance in our data.

The Vitoratou & Garcia-Rosales et al. [10] and the Cogo-Moreira et al. [48] publications were the only studies from this review that consider the impact of the co-occurring disorders on measurement (non)-invariance. Only some studies have considered combining parental and teacher information to enhance measurement reliability.

This systematic review is the first of its kind, looking at measurement invariance using item factor analysis in ADHD, pooling 44 different publications on measurement invariance. Other systematic reviews authored by Gaub & Carlson (1997) [12] and updated by Gershon [68] and Rucklidge (2008, 2010) [13, 69] offer a more comprehensive and detailed overview, considering the factors such as IQ, impairment, comorbidity and interaction with peers. These reviews are very valuable. However, the question remains as to the measurement invariance or non-invariance of the different scales used in the different publications incorporated into these reviews. Measurement invariance is a necessary condition for the comparability of groups.

Parents of the same children report the same information reliably. This is very useful in terms of daily clinical practice. Based on the findings of this review, parents would be interchangeable in terms of the reports of the ADHD symptoms they observe in their children. For now, it is reassuring for clinicians that the information mothers and fathers provide is equally reliable. Teachers also appear to provide reliable information over repeated assessments, which helps monitor ADHD symptoms in routine clinical practice.

Most of the comparisons available from the studies included in this systematic review pointed to both metric and scalar invariances. This supports the current DSM-5-TR [5] diagnostic criteria, where all symptom criteria are considered equal, and there is no consideration of how thresholds may differ. Criterion D of the ADHD diagnostic criteria (DSM-5-TR) [5] referring to impairment across settings may be a proxy for the threshold concept. For example, a very academically orientated child or young person who requires many hours of uninterrupted study sitting down may be more impaired than a young person training for sprint running. Therefore their threshold for specific hyperactivity symptoms might be different.

However, the question remains whether there might be a bias towards publishing and reporting on measurement invariance rather than non-invariance. Measurement non-invariance could potentially introduce more complexity in the nosography of ADHD and enrich it. The concept of loading can be understood intuitively by clinicians familiar with, for example, first-rank psychosis symptoms [70], which were given priority when making a schizophrenia diagnosis for example. Depending on the population considered, the threshold concept could translate into a specific and bespoke symptomatic cut-off. Future studies should include detailed information regarding the direction of bias regarding both loadings and thresholds, including the effect sizes estimations when there is bias [28, 29].

Unfortunately, effect sizes *(and/or standard errors of the estimated parameters involved in measurement invariance assessment)* were not reported in the included studies, which prevented us from exploring measurement non-invariance in a more granular way. Indeed, we could not calculate effect sizes as raw data were not reported. Had this information been available, we could have converted this systematic review into a meta-analysis. This is a significant limitation of our review, and we urge researchers interested in measurement invariance to calculate and report effect sizes whenever measurement non-invariance is established.

The reporting of gender/sex constitutes another limitation in terms of the availability of data. Authors are mindful that the information available is based on reported gender. As our understanding of gender has evolved over the years, there will need to be consideration of the trans and non-binary populations. Therefore, primary studies should incorporate a broader and more updated understanding of gender both in children and young people as well as informants, be they parents, teachers or others, in their data collection and subsequent analyses. In addition, further consideration needs to be given to the value of self-report, in line with the findings by Slobodin et al. [71], where self-reports were associated with parent and teacher reports with a mild to moderate correlation, children self-report of academic-related functioning was associated with continuous performance test performance. This should be the focus of a systematic review in future.

Methodologically, there is much heterogeneity in the model used to fit the scale: unidimensional, using two factors, and the bifactor model. The use of different models impacts the outcomes of the comparisons being made. Understandably, authors are more likely to select the model with the best fit before the likelihood of establishing measurement invariance is enhanced. A more specific focus on the ADHD symptom criteria using a two-dimensional or a bifactor model for consistency. In several studies in this systematic review, different scales incorporate multiple dimensions of other comorbid disorders or exclude some ADHD symptom criteria and/or HI altogether. There was a mixture of populations from across the world, both clinical and non-clinical, which is both a limitation and a strength of this review, particularly regarding teacher information.

In addition, using dependent and independent samples could have yielded slightly different results, for example, in Gomez, 2010 [40] regarding equivalency between mother and father ratings in independent samples. There is definite clinical value in using dependent samples, especially over time, as symptoms are being monitored repeatedly using scales and with the same teachers assessing the young people over time as they progress in a given school, for example.

Ultimately as complexity increases, there needs to be a way of amalgamating information. Clinicians triangulate information and assess impairment to arrive at a diagnostic conclusion informing treatment. According to Garcia-Rosales & Vitoratou et al. [11], parents and teachers appear to be providing fundamentally different types of information, which resonates with the experienced mental health practitioner. The algorithms provided by Valo & Tannock [67] may be a starting point to guide clinicians. There is a further need to develop literature around the combination of parental and teacher information using the ‘or’ (one given symptom criterion is endorsed by either parents or teachers) and ‘and’ (one given symptom criterion is endorsed by both parents and teachers) rules [67] so that the gap is bridged between research and day-to-day clinical practice where the amalgamation of the information is the norm.

In the same way that the advances of statistics have enabled us to start answering the question of measurement invariance and non-invariance in different scales used in ADHD, the use of computer algorithms to pull various sources of information together might be the new frontier for the assessment, diagnosis and monitoring of ADHD. We might be at the inception of a staging model for ADHD, mirroring the model initially developed for cancer treatment [72], which subsequently inspired the one being developed for schizophrenia spectrum disorders [73, 74]. In cancer diagnosis, staging is critical in informing treatment and prognosis. The stages describe the extension of the cancer using the TNM staging system (T for tumour describing the size of the tumour, N for lymph nodes, and M for metastases. The staging directly informs of the treatment. The clinical staging model for psychosis spans stages 0 to 4; 0: at-risk asymptomatic; 1: would be non-specific symptoms or attenuated syndrome; 2: would be a full-threshold disorder, 3: recurrent and persistent illnesses, and 4: unremitting illnesses. These clinical characteristics would be combined with validated biomarkers [75]. Regarding ADHD, such a model could be conceived adjusting for co-morbidity, gender, age and informant regarding assessing the symptoms and potentially incorporating validated biomarkers. This hypothetical model would help index severity and address early the very frequent co-morbidity in ADHD early.

This systematic review should be complemented in the future by an update and a potential focus on other sources of invariance such as ethnicity, country, IQ, race, and language and possibly an update on gender depending on data availability.

## Supporting information

S1 File Search algorithm. (DOCX)

S2 File List of all studies included in the systematic review (underlined articles correspond to the articles added when the search was updated). (DOCX)

S3 File Data availability statement. (DOCX)

S1 Table Excluded articles and reason for exclusion. (DOCX)

S2 Table List of all included studies and their characteristics. (DOCX)

S3 Table Summary table of the age invariance publications (total of 24 tests) with the number of comparisons depending on informant (parents or teachers). (DOCX)

S4 Table Measurement (Non)-Invariance assessment: Younger (Y; less than 10 years old) versus older (O; 11 years old and older) according to parents. Where there is bias the direction of the bias is specified along the number of comparisons. Tables are elaborated based on the available and reported data.
(DOCX)

S5 Table Measurement (Non)-Invariance assessment: Younger (Y; less than 10 years old) versus older (O; 11 years old and older) according to teachers. Where there is bias the direction of the bias is specified along the number of comparisons.
(DOCX)

S6 Table Summary table of the temporal (longitudinal) invariance publications (total of 14 tests) with the number of comparisons depending on informant (parents or teachers). (DOCX)

S7 Table Measurement (Non)-Invariance assessment: Repeated assessments according to parents. Where there is bias the direction of the bias is specified along the number of comparisons.
(DOCX)

S8 Table Measurement (Non)-Invariance assessment: Repeated assessments according to teachers. Where there is bias the direction of the bias is specified along the number of comparisons.
(DOCX)

S1 Checklist PRISMA 2020 check list. (DOCX)
