# Supplementary material for: Measurement invariance of Attention Deficit/Hyperactivity Disorder symptom criteria as rated by parents and teachers in children and adolescents: A systematic review
Source: PLoS One. 2024 Feb 23;19(2):e0293677. doi: 10.1371/journal.pone.0293677 (PMC10889893; doi:10.1371/journal.pone.0293677)
Supplement: S1 File — (DOCX) [file pone.0293677.s001.docx]

## *S1. Search Algorithm*

- ADHD or ADDH or hyperactiv* or attention deficit or attention-deficit or hyperkinetic syndrome or hyperkinetic disorder).mp. [mp=title, abstract, original title, name of substance word, subject heading word, floating sub-heading word, keyword heading word, organism supplementary concept word, protocol supplementary concept word, rare disease supplementary concept word, unique identifier, synonyms]
- symptom*.mp.
- (parent* or home or father or mother).mp. [mp=title, abstract, original title, name of substance word, subject heading word, floating sub-heading word, keyword heading word, organism supplementary concept word, protocol supplementary concept word, rare disease supplementary concept word, unique identifier, synonyms]
- (teacher* or school).mp. [mp=title, abstract, original title, name of substance word, subject heading word, floating sub-heading word, keyword heading word, organism supplementary concept word, protocol supplementary concept word, rare disease supplementary concept word, unique identifier, synonyms]
- IFA or item factor analysis or item-factor analysis or differential item or differential-item or DIF or measurement invarian* or factorial invarian* or measurement equivalen* or item bias or multiple group confirmatory factor analysis or multiple group CFA or multiple indicators multiple causes or MIMIC or invarian* or temporal invarian*).mp. [mp=title, abstract, original title, name of substance word, subject heading word, floating sub-heading word, keyword heading word, organism supplementary concept word, protocol supplementary concept word, rare disease supplementary concept word, unique identifier, synonyms]

For each database, we carried out 3 searches using the above combining 1, 2, 3 and 5 for parent/home searches, 1, 2, 3 and 4 for teacher/school searches and all 5 search terms for parent/home and teacher/school information combined.
