# Supplementary material for: Measurement invariance of Attention Deficit/Hyperactivity Disorder symptom criteria as rated by parents and teachers in children and adolescents: A systematic review
Source: PLoS One. 2024 Feb 23;19(2):e0293677. doi: 10.1371/journal.pone.0293677 (PMC10889893; doi:10.1371/journal.pone.0293677)
Supplement: S2 File — (DOCX) [file pone.0293677.s002.docx]

S2 List of all studies included in the systematic review (underlined articles correspond to the articles added when the search was updated)

**Included articles:**

1. Arias, V. B., Ponce, F. P., Martínez-Molina, A., Arias, B., & Núñez, D. (2016). General and specific attention-deficit/hyperactivity disorder factors of children 4 to 6 years of age: An exploratory structural equation modeling approach to assessing symptom multidimensionality. Journal of Abnormal Psychology, 125(1), 125.
2. Başay Ö, Çiftçi E, Becker SP, Burns GL. Validity of sluggish cognitive tempo in Turkish children and adolescents. Child Psychiatry & Human Development. 2021 Apr;52:191-9.
3. Becker SP, Burns GL, Schmitt AP, Epstein JN, Tamm L. Toward establishing a standard symptom set for assessing sluggish cognitive tempo in children: Evidence from teacher ratings in a community sample. Assessment. 2019 Sep;26(6):1128-41.
4. Beiser M, Dion R, Gotowiec A. The structure of attention-deficit and hyperactivity symptoms among native and non-native elementary school children. Journal of Abnormal Child Psychology. 2000 Oct;28:425-37.
5. Burns GL, Becker SP, Servera M, Bernad MD, García-Banda G. Sluggish cognitive tempo and attention-deficit/hyperactivity disorder (ADHD) inattention in the home and school contexts: Parent and teacher invariance and cross-setting validity. Psychological Assessment. 2017 Feb;29(2):209.
6. Burns GL, Servera M, del Mar Bernad M, Carrillo JM, Geiser C. Ratings of ADHD symptoms and academic impairment by mothers, fathers, teachers, and aides: construct validity within and across settings as well as occasions. Psychological Assessment. 2014 Dec;26(4):1247.
7. Burns GL, Walsh JA, Gomez R, Hafetz N. Measurement and structural invariance of parent ratings of ADHD and ODD symptoms across gender for American and Malaysian children. Psychological Assessment. 2006 Dec;18(4):452.
8. Burns GL, Walsh JA, Patterson DR, Holte CS, Sommers-Flanagan R, Parker CM. Internal validity of the disruptive behavior disorder symptoms: Implications from parent ratings for a dimensional approach to symptom validity. Journal of Abnormal Child Psychology. 1997 Aug;25:307-19.
9. Burns GL, Walsh JA, Servera M, Lorenzo-Seva U, Cardo E, Rodríguez-Fornells A. Construct validity of ADHD/ODD rating scales: Recommendations for the evaluation of forthcoming DSM-V ADHD/ODD scales. Journal of abnormal child psychology. 2013 Jan;41:15-26.
10. Burns GL, Desmul C, Walsh JA, Silpakit C, Ussahawanitchakit P. A multitrait (ADHD–IN, ADHD–HI, ODD toward adults, academic and social competence) by multisource (mothers and fathers) evaluation of the invariance and convergent/discriminant validity of the Child and Adolescent Disruptive Behavior Inventory with Thai adolescents. Psychological Assessment. 2009 Dec;21(4):635.
11. Caci Teacher Ratings of the ADHD-RS IV in a Community Sample: Results From the ChiP-ARD Study."
12. Cogo-Moreira H, Lúcio PS, Swardfager W, Gadelha A, Mari JD, Miguel EC, Rohde LA, Salum GA. Comparability of an ADHD Latent Trait Between Groups: Disentangling True Between-Group Differences From Measurement Problems. Journal of attention disorders. 2019 May;23(7):712-20.
13. Collett BR, Crowley SL, Gimpel GA, Greenson JN. The factor structure of DSM-IV attention deficit-hyperactivity symptoms: A confirmatory factor analysis of the ADHD-SRS. Journal of Psychoeducational Assessment. 2000 Dec;18(4):361-73.
14. de Moura MA, Leonard Burns G. Oppositional defiant behavior toward adults and oppositional defiant behavior toward other children: evidence for two separate constructs with mothers’ and fathers’ ratings of Brazilian children. Journal of Child Psychology and Psychiatry. 2010 Jan;51(1):23-30.
15. de Zeeuw EL, van Beijsterveldt CE, Lubke GH, Glasner TJ, Boomsma DI. Childhood ODD and ADHD behavior: The effect of classroom sharing, gender, teacher gender and their interactions. Behavior Genetics. 2015 Jul;45:394-408.
16. Dobrean A, Păsărelu CR, Balazsi R, Predescu E. Measurement invariance of the ADHD rating scale–IV home and school versions across age, gender, clinical status, and informant. Assessment. 2021 Jan;28(1):86-99.
17. DuPaul GJ, Fu Q, Anastopoulos AD, Reid R, Power TJ. ADHD parent and teacher symptom ratings: Differential item functioning across gender, age, race, and ethnicity. Journal of Abnormal Child Psychology. 2020 May;48:679-91.
18. DuPaul GJ, Reid R, Anastopoulos AD, Lambert MC, Watkins MW, Power TJ. Parent and teacher ratings of attention-deficit/hyperactivity disorder symptoms: Factor structure and normative data. Psychological Assessment. 2016 Feb;28(2):214.
19. Duncan L, Smith S, Wang L, Halladay J. Development and psychometric evaluation of a teacher version of the Ontario child health study emotional behavioural scales (OCHS-EBS-T) for measuring selected DSM-5 disorders in elementary school-aged children. Psychiatry Research. 2022 Jun 1;312:114574.
20. Fumeaux P, Mercier C, Roche S, Iwaz J, Stéphan P, Revol O. Validation of the French version of Conners’ parent rating scale–revised, short form in ADHD-diagnosed children and comparison with control children. Journal of attention disorders. 2021 Jan;25(1):124-33.
21. Gomez R. Testing gender differential item functioning for ordinal and binary scored parent rated ADHD symptoms. Personality and Individual Differences. 2007 Mar 1;42(4):733-42.
22. Gomez R. Invariance of parent ratings of the ADHD symptoms in Australian and Malaysian, and North European Australian and Malay Malaysia Children: A mean and covariance structures analysis approach. Journal of Attention Disorders. 2009 Mar;12(5):422-33.
23. Gomez R. Equivalency for father and mother ratings of the ADHD symptoms. Journal of abnormal child psychology. 2010 Apr;38:303-14.
24. Gomez R. Parent ratings of ADHD symptoms: Generalized partial credit model analysis of differential item functioning across gender. Journal of Attention Disorders. 2012 May;16(4):276-83.
25. Gomez R, Vance A. Parent ratings of ADHD symptoms: Differential symptom functioning across Malaysian Malay and Chinese children. Journal of Abnormal Child Psychology. 2008 Aug;36:955-67.
26. Gomez R, Vance A, Stavropoulos V. Test-retest measurement invariance of clinic referred children’s ADHD symptoms. Journal of Psychopathology and Behavioral Assessment. 2018 Jun;40:194-205.
27. Hall CL, Guo B, Valentine AZ, Groom MJ, Daley D, Sayal K, Hollis C. The validity of the SNAP-IV in children displaying ADHD symptoms. Assessment. 2020 Sep;27(6):1258-71.
28. Hillemeier MM, Foster EM, Heinrichs B, Heier B, Conduct Problems Prevention Research Group. Racial differences in parental reports of attention-deficit/hyperactivity disorder behaviors. Journal of Developmental and Behavioral Pediatrics. 2007 Oct;28(5):353.
29. Jungersen CM, Lonigan CJ. Do parent and teacher ratings of ADHD reflect the same constructs? A measurement invariance analysis. Journal of Psychopathology and Behavioral Assessment. 2021 Dec;43(4):778-92.
30. Khadka G, Burns GL. A measurement framework to determine the construct validity of ADHD/ODD rating scales: Additional evaluations of the Child and Adolescent Disruptive Behavior Inventory. Journal of Psychopathology and Behavioral Assessment. 2013 Sep;35:283-92.
31. Krakowski AD, Cost KT, Szatmari P, Anagnostou E, Crosbie J, Schachar R, Duku E, Georgiades S, Ayub M, Kelley E, Nicolson R. Characterizing the ASD–ADHD phenotype: Measurement structure and invariance in a clinical sample. Journal of Child Psychology and Psychiatry. 2022 Dec;63(12):1534-43.
32. King KM, Luk JW, Witkiewitz K, Racz S, McMahon RJ, Wu J, Conduct Problems Prevention Research Group. Externalizing behavior across childhood as reported by parents and teachers: A partial measurement invariance model. Assessment. 2018 Sep;25(6):744-58.
33. Leopold DR, Christopher ME, Olson RK, Petrill SA, Willcutt EG. Invariance of ADHD symptoms across sex and age: A latent analysis of ADHD and impairment ratings from early childhood into adolescence. Journal of abnormal child psychology. 2019 Jan 15;47:21-34.
34. Lúcio PS, Eid M, Cogo-Moreira H, Puglisi ML, Polanczyk GV. Investigating the Measurement Invariance and Method-Trait Effects of Parent and Teacher SNAP-IV Ratings of Preschool Children. Child Psychiatry & Human Development. 2022 Jun;53(3):489-501.
35. MacDonald B, Pennington BF, Willcutt EG, Dmitrieva J, Samuelsson S, Byrne B, Olson RK. Cross-country differences in parental reporting of symptoms of ADHD. Journal of cross-cultural psychology. 2019 Jul;50(6):806-24.
36. Makransky G, Bilenberg N. Psychometric properties of the parent and teacher ADHD Rating Scale (ADHD-RS) measurement invariance across gender, age, and informant. Assessment. 2014 Dec;21(6):694-705.
37. Narad ME, Garner AA, Peugh JL, Tamm L, Antonini TN, Kingery KM. & Epstein, JN (2015). Parent–teacher agreement on ADHD symptoms across development. Psychological Assessment.;27(1):239
38. Preszler J, Burns GL. Network analysis of ADHD and ODD symptoms: Novel insights or redundant findings with the latent variable model?. Journal of Abnormal Child Psychology. 2019 Oct 15;47:1599-610.
39. Preszler J, Burns GL, Becker SP, Servera M. Multisource longitudinal network and latent variable model analyses of ADHD symptoms in children. Journal of Clinical Child & Adolescent Psychology. 2022 Mar 4;51(2):211-8.
40. Rodenacker K, Hautmann C, Görtz-Dorten A, Döpfner M. Bifactor models show a superior model fit: Examination of the factorial validity of parent-reported and self-reported symptoms of attention-deficit/hyperactivity disorders in children and adolescents. Psychopathology. 2016;49(1):31-9.
41. Toplak ME, Sorge GB, Flora DB, Chen W, Banaschewski T, Buitelaar J, Ebstein R, Eisenberg J, Franke B, Gill M, Miranda A. The hierarchical factor model of ADHD: invariant across age and national groupings?. Journal of Child Psychology and Psychiatry. 2012 Mar;53(3):292-303.
42. Trejo S, Andaverde-Vega AA, Villalobos-Gallegos L, Swanson JM, Salum GA. Factor structure, measurement invariance, and scoring practices of the strengths and weaknesses of ADHD–symptoms and normal behavior. Psychological Assessment. 2022 Dec 1.
43. Vitoratou S, Garcia‐Rosales A, Banaschewski T, Sonuga‐Barke E, Buitelaar J, Oades RD, Rothenberger A, Steinhausen HC, Taylor E, Faraone SV, Chen W. Is the endorsement of the Attention Deficit Hyperactivity Disorder symptom criteria ratings influenced by informant assessment, gender, age, and co‐occurring disorders? A measurement invariance study. International Journal of Methods in Psychiatric Research. 2019 Dec;28(4):e1794.
44. Willoughby MT, Pek J, Greenberg MT, Family Life Project Investigators. Parent-reported attention deficit/hyperactivity symptomatology in preschool-aged children: Factor structure, developmental change, and early risk factors. Journal of abnormal child psychology. 2012 Nov;40:1301-12.
