# Supplementary material for: Measurement invariance of Attention Deficit/Hyperactivity Disorder symptom criteria as rated by parents and teachers in children and adolescents: A systematic review
Source: PLoS One. 2024 Feb 23;19(2):e0293677. doi: 10.1371/journal.pone.0293677 (PMC10889893; doi:10.1371/journal.pone.0293677)
Supplement: S3 File — (DOCX) [file pone.0293677.s003.docx]

# *S3 Data availability statement*

The information used for the systematic review was pooled from published articles referenced in appendix A2.
