# Supplementary material for: Measurement invariance of Attention Deficit/Hyperactivity Disorder symptom criteria as rated by parents and teachers in children and adolescents: A systematic review
Source: PLoS One. 2024 Feb 23;19(2):e0293677. doi: 10.1371/journal.pone.0293677 (PMC10889893; doi:10.1371/journal.pone.0293677)
Supplement: S1 Table — (DOCX) [file pone.0293677.s004.docx]

| *Table S1 Excluded Articles and Reason for exclusion* | |
| --- | --- |
| Reference | Reason for Exclusion |
| 1. Arias VB, Aguayo V, Navas P. Validity of DSM-5 oppositional defiant disorder symptoms in children with intellectual disability. International Journal of Environmental Research and Public Health. 2021 Feb;18(4):1977. | Not about ADHD |
| 1. Arnett AB, Pennington BF, Friend A, Willcutt EG, Byrne B, Samuelsson S, Olson RK. The SWAN captures variance at the negative and positive ends of the ADHD symptom dimension. Journal of attention disorders. 2013 Feb;17(2):152-62. | Not about Measurement Invariance |
| 1. Aydin S, Crone MR, Siebelink BM, Vermeiren RR, Numans ME, Westenberg PM. Recognition of anxiety disorders in children: a cross-sectional vignette-based survey among general practitioners. BMJ open. 2020 Apr 1;10(4):e035799. | Not about ADHD |
| 1. Becker SP, Burns GL, Smith ZR, Langberg JM. Sluggish cognitive tempo in adolescents with and without ADHD: Differentiation from adolescent-reported ADHD inattention and unique associations with internalizing domains. Journal of Abnormal Child Psychology. 2020 Mar;48:391-406. | Self report is an exclusion criteria |
| 1. Bhagia, J., Koplin, B. A., & Halldner-Henriksson, L. Clinical correlates of attention-deficit/hyperactivity disorder and autism spectrum disorder. In meeting of the 6th World Congress on ADHD, Vancouver, FL. 2017 Apr | Excluded, as conference abstract, which is part of the exclusion criteria |
| 1. Bindra A, Chowdhary V, Dube SK, Goyal K, Mathur P. Utility of Serum Procalcitonin in Diagnosing Paroxysmal Sympathetic Hyperactivity in Patients with Traumatic Brain Injury. Indian Journal of Critical Care Medicine: Peer-reviewed, Official Publication of Indian Society of Critical Care Medicine. 2021 May;25(5):580. | Not about ADHD |
| 1. Bioulac S, Lode-Kolz K, Micoulaud-Franchi JA, Chalumeau F, Monteyrol PJ, Philip P. Is it attention deficit hyperactivity disorder, sleep disorder breathing… or both?. Sleep Medicine. 2017 Dec 1;40:e37. | Sleep disorder breathing is not the object of this review |
| 1. Bjørklund O, Belsky J, Wichstrøm L, Steinsbekk S. Predictors of eating behavior in middle childhood: A hybrid fixed effects model. Developmental psychology. 2018 Jun;54(6):1099. | Eating behaviour is not the object of the spit |
| 1. Blackman JA. Attention-deficit/hyperactivity disorder in preschoolers: does it exist and should we treat it?. Pediatric Clinics of North America. 1999 Oct 1;46(5):1011-25. | Treatment studying |
| 1. Bréhin C, Cessans C, Monchaud C, Lavit M, Majorel C, Claudet I. A pseudoencephalitis presentation of a pediatric non-intentional intoxication. European journal of paediatric neurology. 2016 May 1;20(3):418-20. | Pseudoencephalitis is not the object of this review |
| 1. Bridger Staatz, C., et al. (2023). "Investigating the factorial structure and measurement invariance of the parent-reported strengths and difficulties questionnaire at 11 years of age from the UK Millennium Cohort Study." European child & adolescent psychiatry. 11. | Excluded as SDQ |
| 1. Bundgaard AK, Bilenberg N, Asmussen J, Jørgensen PM. Disturbed sleep and activity as early signs of ADHD in preschool children. European Psychiatry. 2016 Mar;33(S1):S346-7. | Sleep in ADHD is not the object of this review |
| 1. Burkart S, Armstrong B. ASSOCIATIONS BETWEEN PRESCHOOLERS'BEHAVIORAL DIFFICULTIES AND VARIABILITY IN SLEEP DURATION AND BEDTIME. InSLEEP 2022 Jun 1 (Vol. 45, pp. A86-A87). JOURNALS DEPT, 2001 EVANS RD, CARY, NC 27513 USA: OXFORD UNIV PRESS INC. | Conference abstract |
| 1. Caci, H., Morin, A. J., & Tran, A. (2015). Investigation of a bifactor model of the Strengths and Difficulties Questionnaire. Europ. Child & Adolesc. Psychiatry, 24, 1291-1301. <https://doi.org/10.1007/s00787-015-0679-3> | SDQ is not a specific scale looking at ADHD |
| 1. Caci H, Morin AJ, Tran A. Investigation of a bifactor model of the Strengths and Difficulties Questionnaire. European Child & Adolescent Psychiatry. 2015 Oct;24:1291-301. | Autism study |
| 1. Chang J, Cimino FM, Gossa W. ADHD in children: common questions and answers. American family physician. 2020 Nov 15;102(10):592-602. | Common questions and answers in ADHD are not the object of this review. |
| 1. Chen C, Lidstone D, Crocetti D, Mostofsky SH, Nebel MB. Increased interhemispheric somatomotor functional connectivity and mirror overflow in ADHD. NeuroImage: Clinical. 2021 Jan 1;31:102759. | Neuroimaging in ADHD is not the object of this review. |
| 1. Chiorri C, Hall J, Casely-Hayford J, Malmberg LE. Evaluating measurement invariance between parents using the Strengths and Difficulties Questionnaire (SDQ). Assessment. 2016 Feb;23(1):63-74. | SDQ is not a specific scale looking at ADHD |
| 1. Cook NE, Sapigao RG, Silverberg ND, Maxwell BA, Zafonte R, Berkner PD, Iverson GL. Attention-deficit/hyperactivity disorder mimics the post-concussion syndrome in adolescents. Frontiers in pediatrics. 2020 Feb 5;8:2. | Irrelevant |
| 1. Cortese, S. Sleep disturbances associated with attention deficit-hyperactivity disorder. Developmental Medicine and Child Neurology. 2011 Jun 3): 5-6. | Irrelevant, about sleep in ADHD |
| 1. Cortese, S., Konofal, E., Lecendreux, M., Arnulf, I., Mouren, M. C., Darra, F., & Bernardina, B. D. (). Restless legs syndrome and attention-deficit/hyperactivity disorder: a review of the literature. Sleep. 2005 Aug;28(8):1007-1013 | Restless leg syndrome is not the object of this review. |
| 1. d'Acremont, M., & Van der Linden, M. (). Confirmatory factor analysis of the Strengths and Difficulties Questionnaire in a community sample of French-speaking adolescents. European Journal of Psychological Assessment. 2008 Jan 24(1): 1-8. | SDQ is not a specific scale looking at ADHD |
| 1. Deutz MH, Shi Q, Vossen HG, Huijding J, Prinzie P, Deković M, van Baar AL, Woltering S. Evaluation of the Strengths and Difficulties Questionnaire-Dysregulation Profile (SDQ-DP). Psychological Assessment. 2018 Sep;30(9):1174. | SDQ-DP is not a specific scale looking at ADHD |
| 1. Suárez AD. PEDIATRÍA INTEGRAL Nº10–DICIEMBRE 2012 Tics en pediatría. | Tics are not the object of this review. |
| 1. Donfrancesco R, Di Trani M, Gregori P, Auguanno G, Melegari MG, Zaninotto S, Luby J. Attention-deficit/hyperactivity disorder and alexithymia: a pilot study. ADHD Attention Deficit and Hyperactivity Disorders. 2013 Dec;5:361-7. | Alexithymia is not the object of this review. |
| 1. Duinhof EL, Lek KM, De Looze ME, Cosma A, Mazur J, Gobina I, Wüstner A, Vollebergh WA, Stevens GW. Revising the self-report strengths and difficulties questionnaire for cross-country comparisons of adolescent mental health problems: the SDQ-R. Epidemiology and psychiatric sciences. 2020;29:e35. | SDQ-R is not a specific scale looking at ADHD |
| 1. Eadeh HM, Breaux R, Langberg JM, Nikolas MA, Becker SP. Multigroup multilevel structure of the child and parent versions of the Positive and Negative Affect Schedule (PANAS) in adolescents with and without ADHD. Psychological Assessment. 2020 Apr;32(4):374. | PANAS is not a specific scale looking at ADHD |
| 1. Endres D, Prüss H, Dressing A, Schneider J, Feige B, Schweizer T, Venhoff N, Nickel K, Meixensberger S, Matysik M, Maier SJ. Psychiatric manifestation of anti-LGI1 encephalitis. Brain sciences. 2020 Jun 16;10(6):375. | Encephalitis is not the object of this review. |
| 1. Etkin P, Ibáñez MI, Ortet G, Mezquita L. Longitudinal Associations Between the Five-Factor Model of Personality and The Bi-Factor Model of Psychopathology: Continuity, Pathoplasty and Complication Effects in Adolescents. Journal of Psychopathology and Behavioral Assessment. 2022 Jun;44(2):405-17. | Does not look at ADHD specific items. |
| 1. Evans SC, Bonadio FT, Bearman SK, Ugueto AM, Chorpita BF, Weisz JR. Assessing the irritable and defiant dimensions of youth oppositional behavior using CBCL and YSR items. Journal of Clinical Child & Adolescent Psychology. 2020 Nov 1;49(6):804-19. | Oppositional behaviour is not the object of this review. |
| 1. Fauteux, A. A., et al. Long-term impacts of kawasaki disease on child development: A Montreal pilot study on cognition, behavior, and electroencephalography markers. Cardiology in the Young. 2022 32(Supplement 2): S235. | Kawasaki disease is not the object of this review. |
| 1. F Foli, K. J., & Elsisy, H. Influence, education, and advocacy: The pediatric nurse's role in the evaluation and management of children with central auditory processing disorders. Journal for Specialists in Pediatric Nursing. 2010 Jan;15(1):62-71. | Central auditory processing disorders are not the object of this review. |
| 1. Forbes MK, Magson NR, Rapee RM. Evidence that different types of peer victimization have equivalent associations with transdiagnostic psychopathology in adolescence. Journal of youth and adolescence. 2020 Mar;49:590-604. | Focus on symptom dimensions in a transdiagnostic way in addition to self-report. |
| 1. Frelut ML. Sleep loss, weight gain. INTERNATIONAL JOURNAL OF PEDIATRIC OBESITY. 2010 Jan 1;5:36-7. | Sleep loss and weight gain are not the object of this review. |
| 1. Garcia-Rosales A, Vitoratou S, Chen W. 6.69 WHAT FACTORS INFLUENCE ATTENTION-DEFICIT/HYPERACTIVITY DISORDER RATINGS APART FROM ATTENTION-DEFICIT/HYPERACTIVITY DISORDER TRAITS?. Journal of the American Academy of Child & Adolescent Psychiatry. 2016;10(55):S226. | Conference abstract |
| 1. Garneau M, Laventure M, Temcheff CE. Internal structure and measurement invariance of the Dominic Interactive among Indigenous children in Quebec. Psychological Assessment. 2020 Feb;32(2):170. | Self-report is an exclusion criterion |
| 1. Gokcen C, Yilmaz G, Karadag M. ADHD symptoms persist even when PTSD symptoms progress: An EMDR case report. Dusunen Adam. 2022 Mar 1;35(1):64-8. | Case report |
| 1. Hall CL, Guo B, Valentine AZ, Groom MJ, Daley D, Sayal K, Hollis C. The validity of the Strengths and Difficulties Questionnaire (SDQ) for children with ADHD symptoms. PloS one. 2019 Jun 19;14(6):e0218518. | Excluded as use4 of SDQ |
| 1. Halse M, Steinsbekk S, Hammar Å, Wichstrøm L. Longitudinal relations between impaired executive function and symptoms of psychiatric disorders in childhood. Journal of Child Psychology and Psychiatry. 2022 Dec;63(12):1574-82. | Excluded, does not look at ADHD specific items. |
| 1. Junghänel M, Wand H, Dose C, Thöne AK, Treier AK, Hanisch C, Ritschel A, Kölch M, Lincke L, Roessner V, Kohls G. Validation of a new emotion regulation self-report questionnaire for children. BMC psychiatry. 2022 Dec;22(1):1-5. | Self-report |
| 1. Garrán RP, Llorca-Bofí V, De Ingunza E. P. 633 So many formulations, so little concentration: how many modifications do we need until finding the optimal treatment for childhood ADHD?. European Neuropsychopharmacology. 2020 Nov 1;40:S356. | Treatment |
| 1. Gessa GL, Pani L, Fadda P, Fratta W. Sleep deprivation in the rat: an animal model of mania. European Neuropsychopharmacology. 1995 Jan 1;5:89-93 | Animal study on mania |
| 1. Golomb RG, Mouton-Odum S. Psychological interventions for children with sensory dysregulation. Guilford Publications; 2016 Aug 2. | Piedmont unsanitary, dysregulation or not the objects of this review. |
| 1. Gomez R. Malaysian parent and teacher ratings of the oppositional defiant disorder symptoms: measurement invariance and parent–teacher agreement. Asian Journal of Psychiatry. 2014 Oct 1;11:35-8. | ODD and not ADHD |
| 1. Gomez R. Teacher ratings of ODD symptoms: Measurement equivalence across Malaysian Malay, Chinese and Indian children. Asian Journal of Psychiatry. 2014 Apr 1;8:52-5. | ODD and not ADHD |
| 1. Gómez-Benito J, Van de Vijver FJ, Balluerka N, Caterino L. Cross-cultural and gender differences in ADHD among young adults. Journal of attention disorders. 2019 Jan;23(1):22-31. | Young adults: exclusion criterion |
| 1. Green JG, DeYoung G, Wogan ME, Wolf EJ, Lane KL, Adler LA. Evidence for the reliability and preliminary validity of the Adult ADHD Self‐Report Scale v1. 1 (ASRS v1. 1) Screener in an adolescent community sample. International journal of methods in psychiatric research. 2019 Mar;28(1):e1751. | Self report: exclusion criterion |
| 1. Gustafsson BM, Danielsson H, Granlund M, Gustafsson PA, Proczkowska M. Hyperactivity precedes conduct problems in preschool children: a longitudinal study. BJPsych open. 2018 Jul;4(4):186-91. | Hyperactivity predicting conduct problems is not the objects of this study. |
| 1. Hall, C. L., et al. (2019). "The validity of the Strengths and Difficulties Questionnaire (SDQ) for children with ADHD symptoms." PLoS ONE [Electronic Resource] 14(6) (no pagination). | Excluded as SDQ |
| 1. Halse, M., et al. (2022). "Longitudinal relations between impaired executive function and symptoms of psychiatric disorders in childhood." Journal of child psychology and psychiatry, and allied disciplines 63(12): 1574-1582. | Excluded, does not look at ADHD specific items. |
| 1. Herzhoff K. Gender Differences in Youth Externalizing Comorbidity (Doctoral dissertation, Northwestern University). | Conference abstract |
| 1. Hoffmann MD, Lang JJ, Guerrero MD, Cameron JD, Goldfield GS, Orpana HM, De Groh M. Evaluating the psychometric properties of the parent-rated Strengths and Difficulties Questionnaire in a nationally representative sample of Canadian children and adolescents aged 6 to 17 years. Health Rep. 2020 Aug 1;31:13-20. | SDQ is not a specific scale looking at ADHD |
| 1. Hųjgaard D, Mortensen EL, Ivarsson T, Hybel KA, Skarphedinsson GA, Nissen JB, Valderhaug R, Dahl K, Weidle B, Thomsen PH. Symptom Dimensions and Clinical Correlates of OCD in a Large Sample of Children and Adolescents. In65th Annual Meeting 2018 Oct 24. AACAP. | OCD not ADHD |
| 1. Højgaard DR, Mortensen EL, Ivarsson T, Hybel K, Skarphedinsson G, Nissen JB, Valderhaug R, Dahl K, Weidle B, Torp NC, Grados M. Structure and clinical correlates of obsessive–compulsive symptoms in a large sample of children and adolescents: a factor analytic study across five nations. European child & adolescent psychiatry. 2017 Mar;26:281-91. | OCD not ADHD |
| 1. Houghton S, Hunter SC, Trewin T, Glasgow K, Carroll A. The multidimensional anxiety scale for children: A further validation with Australian adolescents with and without ADHD. Journal of Attention Disorders. 2014 Jul;18(5):402-11. | Anxiety scale, not ADHD scale |
| 1. Hygen BW, Skalická V, Stenseng F, Belsky J, Steinsbekk S, Wichstrøm L. The co‐occurrence between symptoms of internet gaming disorder and psychiatric disorders in childhood and adolescence: prospective relations or common causes?. Journal of Child Psychology and Psychiatry. 2020 Aug;61(8):890-8. | Gaming disorders are not the object of this review. |
| 1. Iwata N, Kumagai R, Saeki I. Do mothers and fathers assess their children's behavioral problems in the same way as do their children? an IRT investigation on the strengths and difficulties questionnaire. Japanese Psychological Research. 2020 Apr;62(2):87-100. | SDQ is not a specific scale looking at ADHD |
| 1. Janitza S, Klipker K, Hölling H. Age-specific norms and validation of the German SDQ parent version based on a nationally representative sample (KiGGS). European Child & Adolescent Psychiatry. 2020 Feb;29:123-36. | SDQ is not a specific scale looking at ADHD |
| 1. Junghanel, M., et al. (2022). "Validation of a new emotion regulation self-report questionnaire for children." BMC Psychiatry Vol 22 2022, ArtID 820 22. | Excluded as self report. |
| 1. Kase BE, Rommelse N, Chen Q, Li L, Andersson A, Du Rietz E, Vos M, Cortese S, Larsson H, Hartman CA. Longitudinal associations between symptoms of ADHD and BMI from late childhood to early adulthood. Pediatrics. 2021 Jun 1;147(6). | Associations between ADHD symptoms and BMI are not the object of this review. |
| 1. Kelly D, Forney J, Parker-Fisher S, Jones M. Evaluating and managing attention deficit disorder in children who are deaf or hard of hearing. American Annals of the Deaf. 1993;138(4):349-57. | The evaluation or management of ADHD in hearing impaired populations is not the object of this review. |
| 1. Kenney C, Kuo SH, Jimenez-Shahed J. Tourette's syndrome. American Family Physician. 2008 Mar 1;77(5):651-8. | Tourette’s is not the object of this review. |
| 1. Klein B, Damiani‐Taraba G, Koster A, Campbell J, Scholz C. Diagnosing attention‐deficit hyperactivity disorder (ADHD) in children involved with child protection services: are current diagnostic guidelines acceptable for vulnerable populations?. Child: care, health and development. 2015 Mar;41(2):178-85. | Irrelevant, about sleep and ADHD |
| 1. Krakowski A, Cost K, Szatmari P, Anagnostou E, Crosbie J, Schachar R, Duku E, Georgiades S, Barnett-Tapia C. 6.25 Characterizing the Autism Spectrum Disorder–ADHD Phenotype: Measurement Structure and Invariance in a Clinical Sample. Journal of the American Academy of Child & Adolescent Psychiatry. 2021 Oct 1;60(10):S166. | Conference abstract |
| 1. Kumar, P., et al. "Childhood bipolar disorder masquerading as Atten.-Deficit/Hyperactivity Disorder with episodic obsessive compulsive disorder: A case report with a review of the literature. 2017 Apr ADHD Atten. Deficit and Hyperactivity Dis.9(1 Supplement):S17-S18. | Case report |
| 1. Lavigne JV, Bryant FB, Hopkins J, Gouze KR. Dimensions of oppositional defiant disorder in young children: Model comparisons, gender and longitudinal invariance. Journal of Abnormal Child Psychology. 2015 Apr;43:423-39. | Oppositional Defiant Disorder is not the object of this review. |
| 1. Lonigan CJ, Lerner MD, Goodrich JM, Farrington AL, Allan DM. Executive function of Spanish-speaking language-minority preschoolers: Structure and relations with early literacy skills and behavioral outcomes. Journal of experimental child psychology. 2016 Apr 1;144:46-65. | Executive function is not the object of this review. |
| 1. López-Romero L, Romero E, Colins OF, Andershed H, Hare RD, Salekin RT. Proposed Specifiers for Conduct Disorder (PSCD): Preliminary validation of the parent version in a Spanish sample of preschoolers. Psychological assessment. 2019 Nov;31(11):1357. | Specifiers of conduct disorders are not the object of this review. |
| 1. Lougy RA, DeRuvo SL, Rosenthal D, editors. Teaching young children with ADHD: Successful strategies and practical interventions for preK-3. Corwin Press; 2007 Mar 28. | The teaching of young children with ADHD is not the object of this review. |
| 1. March JS, Parker JD, Sullivan K, Stallings P, Conners CK. The Multidimensional Anxiety Scale for Children (MASC): factor structure, reliability, and validity. Journal of the American academy of child & adolescent psychiatry. 1997 Apr 1;36(4):554-65. | Anxiety scale |
| 1. Martsenkovsky I, Martsenkovska I. Inattention, hyperactivity and impulsivity as factors in children of academic unsuccess. InEUROPEAN CHILD & ADOLESCENT PSYCHIATRY 2011 Jun 1 (Vol. 20, No. 1, pp. S123-S123). 233 SPRING ST, NEW YORK, NY 10013 USA: SPRINGER. | Academic unsuccess in ADHD is not the object of this review. |
| 1. Martsenkovskyi D, Martsenkovsky I. Relationship between early childhood trauma and attention deficit hyperactivity disorder. InEUROPEAN CHILD & ADOLESCENT PSYCHIATRY 2013 Jul 1 (Vol. 22, pp. S258-S258). 233 SPRING ST, NEW YORK, NY 10013 USA: SPRINGER. | Early childhood trauma and ADHD are not the object of this review. |
| 1. Martsenkovskyi, D. and I. Martsenkovsky (). Children mental health in refugee families: Childhood abuse as a cause of ADHD. 2015 May [ADHD Attention Deficit and Hyperactivity Disorders](https://link.springer.com/journal/12402). 1): S70. | Child abuse as a cause of ADHD is not the object of this review. |
| 1. Mautone JA, Pendergast LL, Cassano M, Blum NJ, Power TJ. Behavioral health screening: validation of a strength-based approach. Journal of Developmental & Behavioral Pediatrics. 2020 Oct 1;41(8):587-95. | Screening questionnaire, not ADHD specific |
| 1. Mautone JA, Power T, Blum NJ, Pendergast L, Watkins M, Cassano M, Koshy A. 4.50 THE BEHAVIORAL HEALTH CHECKLIST: CULTURALLY SENSITIVE AND STRENGTH-BASED SCREENING IN PEDIATRIC PRIMARY CARE. Journal of the American Academy of Child & Adolescent Psychiatry. 2016;10(55):S178-9. | Conference Abstract |
| 1. McElroy E, Shevlin M, Murphy J, McBride O. Co-occurring internalizing and externalizing psychopathology in childhood and adolescence: a network approach. European child & adolescent psychiatry. 2018 Nov;27:1449-57. | Not sufficiently specific |
| 1. Monastra VJ. Unlocking the potential of patients with ADHD: A model for clinical practice. American Psychological Association; 2008. | A model for clinical practice in ADHD is not the object of this review. |
| 1. Morton H. Development and Validation of the Assessment of Bullying Experiences Questionnaire: A Data-Driven Measure for Bullying Assessment in Youth with Autism Spectrum Disorder(Doctoral dissertation, State University of New York at Binghamton). | Not ADHD specific, in ASD population |
| 1. Murray AL, Booth T, Eisner M, Ribeaud D, McKenzie K, Murray G. An analysis of response shifts in teacher reports associated with the use of a universal school-based intervention to reduce externalising behaviour. Prevention Science. 2019 Nov;20:1265-73. | Not ADHD specific. Intervention study. |
| 1. Murray AL, Speyer LG, Hall HA, Valdebenito S, Hughes C. Teacher versus parent informant measurement invariance of the Strengths and Difficulties Questionnaire. Journal of Pediatric Psychology. 2021 Nov;46(10):1249-57. | SDQ is not a specific scale looking at ADHD |
| 1. Navalkar N, Pavuluri A, Trasmonte J. Case Report: Transient Global Amnesia in a Child. InANNALS OF NEUROLOGY 2020 Oct 1 (Vol. 88, pp. S149-S150). 111 RIVER ST, HOBOKEN 07030-5774, NJ USA: WILEY. | Case report |
| 1. Normand S, Mikami AY, Savalei V, Guiet J. A Multiple Indicators Multiple Causes (MIMIC) model of friendship quality and comorbidities in children with attention-deficit/hyperactivity disorder. Psychological Assessment. 2020 Jul;32(7):698. | Tangential |
| 1. Numata‐Uematsu Y, Yokoyama H, Sato H, Endo W, Uematsu M, Nara C, Kure S. Attachment Disorder and Early Media Exposure: Neurobehavioral symptoms mimicking autism spectrum disorder. The Journal of Medical Investigation. 2018;65(3.4):280-2. | Attachment disorder and early media exposure are not the object of this review. |
| 1. Ogg J, McMahan MM, Dedrick RF, Mendez LR. Middle school students' willingness to engage in activities with peers with ADHD symptoms: A multiple indicators multiple causes (MIMIC) model. Journal of School Psychology. 2013 Jun 1;51(3):407-20. | Peer engagement is not the object of this review. |
| 1. Ogundele MO. G467 Management of sleep difficulties among a cohort of children with adhd in a scottish local authority. | Sleep and ADHD |
| 1. Opocher E, Bisogno G, Calderone M, Drigo P. Unusual neuroimaging in a young boy with cerebral X-linked adrenoleukodystrophy. Neuropediatrics. 2010 Aug;41(02):66-8. | Not about ADHD. |
| 1. Palmieri PA, Smith GC. Examining the structural validity of the Strengths and Difficulties Questionnaire (SDQ) in a US sample of custodial grandmothers. Psychological assessment. 2007 Jun;19(2):189. | SDQ is not a specific scale looking at ADHD |
| 1. Park JL, Silveira M, Elliott M, Savalei V, Johnston C. Confirmatory factor analysis of the structure of adult ADHD symptoms. Journal of Psychopathology and Behavioral Assessment. 2018 Dec;40:573-85. | Adult population: exclusion criteria |
| 1. Praud JP. Sleep-Disordered Breathing in Children: Which Study and When?. InPEDIATRIC PULMONOLOGY 2019 Jun 1 (Vol. 54, pp. S41-S43). 111 RIVER ST, HOBOKEN 07030-5774, NJ USA: WILEY. | Not about ADHD |
| 1. Preszler J, Burns GL, Litson K, Geiser C, Servera M, Becker SP. How consistent is sluggish cognitive tempo across occasions, sources, and settings? Evidence from latent state–trait modeling. Assessment. 2019 Jan;26(1):99-110. | Latent state-trait modelling in sluggish cognitive tempo. |
| 1. Quinn, D. Differential item function in a rasch-validated emotional dysregulation scale when assessing oppositional defiant disorder in children with ADHD: Implications for clinical practice. 2015 May [ADHD Attention Deficit and Hyperactivity Disorders](https://link.springer.com/journal/12402) 1):S76-S77. | Not ADHD scale looking at diagnostic criteria |
| 1. Rapport MD, Kofler MJ, Coiro MM, Raiker JS, Sarver DE, Alderson RM. Unexpected effects of methylphenidate in attention-deficit/hyperactivity disorder reflect decreases in core/secondary symptoms and physical complaints common to all children. Journal of child and adolescent psychopharmacology. 2008 Jun 1;18(3):237-47. | Irrelevant, about treatment |
| 1. Rivers AS, Winston-Lindeboom P, Ruan-Iu L, Atte T, Tien A, Diamond G. Validation of a parent report on externalizing symptoms scale: A downward extension of the behavioral health screen. School Psychology. 2022 Dec 22. | Excluded, does not look at ADHD specific items based on DSM. |
| 1. Rodriguez‐Seijas C, Gadow KD, Rosen TE, Kim H, Lerner MD, Eaton NR. A transdiagnostic model of psychiatric symptom co‐occurrence and autism spectrum disorder. Autism Research. 2020 Apr;13(4):579-90. | No separation of inattentiveness and hyperactivity |
| 1. Rowland AS, Lesesne CA, Abramowitz AJ. The epidemiology of attention‐deficit/hyperactivity disorder (ADHD): a public health view. Mental retardation and developmental disabilities research reviews. 2002;8(3):162-70. | Epidemiology of ADHD |
| 1. Sáez B, Servera M, Becker SP, Burns GL. Optimal items for assessing sluggish cognitive tempo in children across mother, father, and teacher ratings. Journal of Clinical Child & Adolescent Psychology. 2019 Nov 2;48(6):825-39. | SCT assessment |
| 1. Sarampote CS, Efron LA, Robb AS, Pearl PL, Stein MA. Can stimulant rebound mimic pediatric bipolar disorder?. Journal of Child and Adolescent Psychopharmacology. 2002 Mar 1;12(1):63-7. | Psychopharmacology |
| 1. Scannapieco, M. and K. R. Painter.Attention deficit hyperactivity disorder. Raines JC, editor. Evidence-based practice in school mental health: Addressing DSM-5 disorders in schools. Oxford University Press; 2019 Mar 11:61-90. | Book: exclusion criterion |
| 1. Schonwald, A. ADHD in adolescents: A comprehensive guide. ADHD in adolescents: A comprehensive guide xiii, 300 pp Cham, Switzerland: Springer Nature Switzerland AG; Switzerland; 2020. | Book chapter |
| 1. Schweitzer, J. B., et al. Attention deficit/hyperactivity disorder. 2012; Handbook of Clinical Neurology 106: 391-405. | Book: exclusion criterion |
| 1. Seijas R, Servera M, García-Banda G, Barry CT, Burns GL. Evaluation of a four-item DSM–5 Limited Prosocial Emotions specifier scale within and across settings with Spanish children. Psychological Assessment. 2018 Apr;30(4):474. | Not ADHD specific scale |
| 1. Srifuengfung M, Bussaratid S, Ratta-Apha W, Sanguanpanich N, Hosiri T. Restless legs syndrome in children and adolescents with attention-deficit/hyperactivity disorder: prevalence, mimic conditions, risk factors, and association with functional impairment. Sleep Medicine. 2020 Sep 1;73:117-24. | Restless legs syndrome |
| 1. Staatz CB, Kelly Y, Lacey R, Hardy R. Investigating the factorial structure and measurement invariance of the parent-reported Strengths and Difficulties Questionnaire at 11 years old from the UK Millennium Cohort Study. | Excluded as SCQ |
| 1. Stein MA. A Shared Decision-Making Approach to Evaluating, Sequencing, and Combining Interventions in Teenagers With ADHD. In2020 Virtual Meeting 2020 Oct 19. AACAP. | Treatment |
| 1. Stein, M. A. and M. L. Batshaw. Attention-Deficit/Hyperactivity disorder. 2001 When your child has a disability: The complete sourcebook of daily and medical care, Rev ed. Baltimore, MD, Paul H Brookes Publishing; US: 355-371. | Book: exclusion criterion |
| 1. Steinlechner S, Brüggemann N, Sobottka V, Benthien A, Behn B, Klein C, Schmid G, Lencer R. Restless legs syndrome as a possible predictor for psychiatric disorders in parents of children with ADHD. European archives of psychiatry and clinical neuroscience. 2011 Jun;261:285-91. | Restless legs syndrome in parents of young people with ADHD. |
| 1. Stiles-Shields C, Skelly CL, Mak GZ, Speaker C, Boyd H, O’Brien S, Drossos T. Psychological factors and outcomes in the surgical treatment of pediatric patients with median arcuate ligament syndrome. Journal of Pediatric Gastroenterology and Nutrition. 2018 Jun 1;66(6):866-71. | Surgery |
| 1. Stoddard FJ, Usher CT, Abrams AN. Psychopharmacology in pediatric critical care. Child and Adolescent Psychiatric Clinics. 2006 Jul 1;15(3):611-55. | Psychopharmacology |
| 1. Sulestrowska H. Personality Disorders in Children: Problems in Diagnosis and Definition. International Journal of Mental Health. 1975 Dec 1;4(4):36-42. | Personality disorders |
| 1. Thöne AK, Junghänel M, Görtz-Dorten A, Dose C, Hautmann C, Jendreizik LT, Treier AK, Vetter P, Von Wirth E, Banaschewski T, Becker K. Disentangling symptoms of externalizing disorders in children using multiple measures and informants. Psychological Assessment. 2021 Nov;33(11):1065. | Five factors of externalizing disorders |
| 1. Thöne AK, Junghänel M, Görtz-Dorten A, Breuer D, del Giudice T, Hanisch C, Hennemann T, Döpfner M. Empirically based dimensions of externalizing symptoms in children and adolescents: a multitrait-multisource approach. Journal of Psychopathology and Behavioral Assessment. 2022 Sep;44(3):844-61. | Does not map onto DSM items. |
| 1. Tijero-Merino B, Gomez-Esteban JC, Zarranz JJ. Tics and Gilles de la Tourette syndrome. Revista de neurologia. 2009 Jan 1;48:S17-20. | Irrelevant |
| 1. Luk ES, Costin J, Tonge BJ, Pantelis C. Attention deficit hyperactivity disorder: anxiety phenomena in children treated with psychostimulant medication for 6 months or more. Australian and New Zealand Journal of Psychiatry. 1999 Jun;33(3):399-406. | Treatment |
| 1. Waxmonsky JG. 4.3 A Smart Design Examining the Impact of CNS Stimulants On the Growth Trajectories of Children With Attention-Deficit/Hyperactivity Disorder. Journal of the American Academy of Child & Adolescent Psychiatry. 2016;10(55):S263-4. | Treatment |
| 1. M Weiss J, A Boss-Williams K. The hyperactive rat: an animal model for bipolar disorder. Current Psychiatry Reviews. 2017 Jun 1;13(2):111-62. | Animal study |
| 1. Wichstrøm L, Penelo E, Rensvik Viddal K, de la Osa N, Ezpeleta L. Explaining the relationship between temperament and symptoms of psychiatric disorders from preschool to middle childhood: hybrid fixed and random effects models of Norwegian and Spanish children. Journal of Child Psychology and Psychiatry. 2018 Mar;59(3):285-95. | Temperament study |
| 1. Suwanee G. An introduction to oppositional defiant disorder and conduct disorder. US Pharm. 2019;44(11):29-32. | Oppositional defiant disorder and conduct disorder publication meant as an introduction to the field. |
| 1. Yao S, Zhang C, Zhu X, Jing X, McWhinnie CM, Abela JR. Measuring adolescent psychopathology: psychometric properties of the self-report strengths and difficulties questionnaire in a sample of Chinese adolescents. Journal of Adolescent Health. 2009 Jul 1;45(1):55-62. | Self report as well asUse of the SDQ which is not an ADHD specific scale |
| 1. Yoo B, Katz J, Bynum D. Serotonin Syndrome from Polypharmacy. In JOURNAL OF THE AMERICAN GERIATRICS SOCIETY 2018 Apr 1 (Vol. 66, pp. S95-S95). 111 RIVER ST, HOBOKEN 07030-5774, NJ USA: WILEY. | Serotonin syndrome |
