# Supplementary material for: Measurement invariance of Attention Deficit/Hyperactivity Disorder symptom criteria as rated by parents and teachers in children and adolescents: A systematic review
Source: PLoS One. 2024 Feb 23;19(2):e0293677. doi: 10.1371/journal.pone.0293677 (PMC10889893; doi:10.1371/journal.pone.0293677)
Supplement: S2 Table — (DOCX) [file pone.0293677.s005.docx]

| *Table S2: List of all included studies and their characteristics* | | | | | | | |
| --- | --- | --- | --- | --- | --- | --- | --- |
| Reference | Study Design | Geographical Location | ADHD Scale used | Male/Female | Clinical vs Community | Age | N |
| 1. Arias, et al. , 2016 | Cross-Sectional | Spain | ADHD questionnaire Teachers | 50.5% female | Community | Mean age boys: 5.1 (SD 0.74)  Mean age girls: 5.2 (SD 0.76) | 650 |
| 1. Başay et al., 2021 | Cross-Sectional | Turkey | Child and Adolescent Behavior Inventory (CADBI) | Community: 60,1% girls  Clinical: 44,5% girls | Community and Clinical | Community: Mean age: 9.86 (SD 2.21)  Clinical: Mean age; 10,61 (SD=2,54) | Community: 762  Clinical: 253 |
| 1. Becker et al., 2019 | Cross-Sectional | Midwestern USA | Child and Adolescent Behavior Inventory (CADBI) | 49.3% female | community | grades 2 to 5 (7-11 years old)  Mean age not provided | 1 349 |
| 1. Beiser et al., 2000 | Cross-Sectional sample of a longitudinal study | Data from the Flower of Tow Soils (FOTS) study, a longitudinal investigation of cognitive development, mental health and academic achievement among Native and non-Native children that took place at 4 geographically disparate sites, two in the US and two in Canada. | Teacher Interview Form (TIF)  Child's Assesssment by a Parent (CAP) | 52% female | Community | Mean age of grade 2 students: 8,4 years old  Mean age of grade 4: 10,4 years old | 1155 Natives and 489 non-Native children |
| 1. Burns et al., 2017 | Cross-sectional | Spain | Child and Adolescent Disruptive Behavior Inventory (CADBI) parent version and teacher version | 47% female | community | 3rd grade children (8-9 years old) | 585 |
| 1. Burns et al., 2014 | Longitudinal, 2 assessments at 6-week interval | Spain | Child and Adolescent Disruptive Behavior Inventory (CADBI) parent and teacher | 46% female |  | 1st grade children (6-7 years old) | 811 |
| 1. Burns et al,, 2006 | Cross-Sectional | American sample from 5 pediatric clinics in Washington, Idaho, Montana and Virginia.  Malaysian sample from 14 elementary schools in Johor Bahru. | Child and Adolescent Disruptive Behavior Inventory (CADBI) parent version  Disruptive Behaviour Questionnaire (DBQ) parent | American clinical sample:  45.4% female  Malaysian Community Sample: 53,1% female | American clinical sample  Malaysian Community Sample | American Sample: Mean age boys: 8.64 (sd 3.65)  Mean age girls: 8.74 (sd 3.67)  Malaysian sample: mean age of boys: 8.88 (SD= 1.64), Mean age of girls 9.02 (SD=1.74) | American clinical sample of 1015 children |
| 1. Burns et al., 1997 | Cross-sectional | US samples 2 community samples Seattle (Washington) and Missoula (Montana) populations and 1 clinical sample from 5 pediatric clinics of 4 Northwestern states. | Child and Adolescent Disruptive Behavior Inventory (CADBI): Parent and Rating Scale | 46.63% female | mixed community and pediatric clinic sample | Mean age 10.36 (sd 4.32) range 2 to 19 | 4019 |
| 1. Burns et al,, 2013 | Cross-sectional | Thailand  Spain | Child and Adolescent Disruptive Behavior Inventory (CADBI) parent and teacher versions | Thai: 61% female  48% female | Community Thai and Spanish samples | Thai: 14.99 (sd 1.77)  Spanish: 8.31 (sd 1.21) | Thai: 872 Spanish: 1749 |
| 1. Burns et al., 2009 | Longitudinal, analyses repeated for each year | Thailand | Child and Adolescent Disruptive Behavior Inventory (CADBI) parent | 63% female, ranging from 61% to 65% | Community | Mean age varied from 14.64 to 15.57 (sd range from 1.61 to 1.77) | Year 1, n= 872  Year 2, n=903  Year 3, n=700  Year 4, n=984 |
| 1. Caci et al., 2016 | Cross-sectional | France | ADHD Rating scale (ADHD-RS), teacher scale | 51.35% female | Community | mean age for girls 10.59 (sd 3.5)  mean age for boys 10.18 (sd 3.32) | 892 |
| 1. Cogo-Moreira et al., 2019 | Cross-sectional | Brazil | DAWBA administered by trained lay interviewer to biological mother  WISC-III | Not available | High-risk subjects and random sample | between 6-14 years of age | 2 299 |
| 1. Collett et al., 2000 | Cross-sectional | Idaho, USA | ADHD Symptoms Rating Scale (ADHD-SRS), parent version | 46% female | Community | Mean age: 8.09 (sd 1.81) | 572 |
| 1. de Zeeuw et al. 2015 | Cross-sectional | Netherlands | Conners' Teacher Rating Scales- Revised (CTRS-R) |  | Netherlands twin register | See next column | 8 611: 7 year olds  8 02: 9 year olds  5 95:12 year olds |
| 1. Dobrean et al., 2021 | Cross-Sectional | Romania | ADHD-RS-IV parent version  ADHD-RS-IV teacher version | 52.6% female | Community and Clinical | Mean age 12.74, sd 2.84 | 1 106 |
| 1. DuPaul et al., 2020 | Cross-sectional, independent samples for teacher and parent ratings. | Metropolitan and non-Metropolitan USA | ADHD Rating Scale-5 Home (ARS-5)  ADHD Rating Scale-5 School versions (ARS-5) | Parent sample: 50.1% female, Mean age = 10.68, sd 3.75  Teacher sample: 50% female | Community | Parent sample: Mean age = 10.68, sd 3.75  Teacher sample: mean age = 11.53, sd 3.54 | Parent sample: 2 079  Teacher sample 1 070 |
| 1. DuPaul et al., 2016 | Cross-sectional, 2 independent samples | USA representative population | ADHD Rating Scale-5 Home Version  ADHD Rating Scale-5 School Version | Parent sample: 50.1% female  Teacher sample: 50% female | Community | Parent sample: Mean age = 10.68, sd 3.75  Teacher sample: mean age = 11.53 | Parent sample: 2 079  Teacher sample: 1 070 |
| 1. Duncan et al., 2022.. | Cross-Sectional | Canadina Child Tax Benefit file as the sampling frame | (Ontario Child Health Study Emotional Behavioural ScalesOCHS) Emotional Behaviour Scales-Teacher Version (OCHS-EBS-T) and Parent/Caregiver Version (OCHS-EBS-P) | 48.3% female | Community | Mena age: 8.54 (2.85) | 3072 |
| 1. Fumeaux et al., 2021 | Cross-sectional case-control study | France | French Lausanne Version Conners Parent RATING Scale-Revised, Short Form (FLV CPRS-R:S) | Case group: 19.4% female  Control group: 50,6% female | Community and Clinical | Case group: 12 (sd=3.3). Case group was on average 8 months younger than the control group | 108 AHD children aged 6-17 versus 794 control aged 6-15 |
| 1. Gomez et al., 2007 | Cross-sectional | Australia | DSM-IV ADHD rating Scale (DARS) parent scale, ordinal ratings | 50.3% female | Community | Mean age boys 8.29 (sd 1.28)  Mean age girls 8.27 (1.81) | 1 475 |
| 1. Gomez et al., 2009. 2009 | Cross-Sectional | Victoria state, Australia and Johor state, Malaysia | Disruptive Behavior Rating Scale (DBRS) parents only focusing on IA and HI | Australian Sample: 48,5% female  Malaysian Sample: 53.1% female | Community | Australian Sample: 8.86 (sd= 1.99)  Malaysian Sample: 8.95 (sd=1.69) | Australian Sample: 783  Malaysian Sample: 928 |
| 1. Gomez et al., 2010 | Cross-Sectional | Australia | Disruptive Behavior Disorders Rating Scale (DBRS) only use IA and HI scales | Father-rated sample: 50.1% female,  Mother-rated sample: 50.1% female, mean age 8.53 (sd 1.84) | Community | Father-rated sample: mean age 8.72 (sd 1.95)  Mother-rated sample: mean age 8.53 (sd 1.84) | Father-rated sample: 367  Mother-rated sample 411 |
| 1. Gomez et al., 2012 | Cross-Sectional | Victoria, Australia | Disruptive Behavior rating scale (DBRS) in parents | 48.5% female | Community | Mean age for boys 8.88 (sd 1.96)  Mean age for girls 8.83 (sd 2.02) | 783 |
| 1. Gomez et al., 2008 | Cross-Sectional | Johor, Malaysia | Disruptive Behavior rating scale (DBRS) in parents | Malay: 55.5% fremale  Chinese: 50.7% female | Community | Mean age Malay 8.33 (sd 1.70)  Mean age Chinese 9.22 (sd 1.59) | 571 Malay and 274 Chinese |
| 1. Gomez et al., 2018 | Cross-Sectional: 2 repeated analyses12 months apart | Melbourne, Australia | Strengths and Weaknesses of ADHD-Symptoms and Normal Behavior Scale (SWAN) in mothers | 22.6% female | Clinical | between 7 and 17 year old | 217 |
| 1. Hall et al., 2020 | Cross-Sectional | England | Swanson, Nolan and Pelham Rating Scale (SNAP-IV) for parents and teachers | 21% female | Clinical | Mean age 9.5 (sd 2.8) | 250 |
| 1. Hillemeier et al., 2007 | Case control study, which is part of the Fast-Track study | North Carolina, Tennessee, Pennsylvania and Washington, USA | Diagnostic Interview Schedule for Children (DISC) in parents | 36% female | High risk cohort based on crime and poverty statistics of their neighbouraood, part of th Fast Track Project. | Not available | 1 070 |
| 1. Jungersen et al., 2021 | Cross-Sectional | North Florida, USA | Strengths and Weaknesses of ADHD symmptoms and Normal Behavior Rating Scale (SWAN), used for parents and teachers.  Conners Teacher's Rating Scale-15 (CTRS-15), used for parents and teachers. | Approximately 1:1 female-male ratio | Community | between 46 and 169 months | 1645 |
| 1. Khadka et al., 2013 | Cross-sectional, 2 independent groups | Thailand | Child and Adolescent Behavior Inventory (CADBI) in mothers and fathers | - group 1: 61% female  - group 2: 62% female | Community | group 1: mean age 14.99 (SD 1.77),  group 2: mean age =15.02 (sd 1.61). | group 1: n=872  group 2: n=983 |
| 1. Krakowski et al., 2022 | Cross-Sectional | Province of Ontario Neurodevelopmental Disorders (POND) Network, Ontario, Canada | Strengths and Weaknesses of ADHD Symptoms and Normal Behaviour Rating Scale (SWAN) for parents | 22% males in ASD sample  25% female in ADHD sample | Clinical diagnosis of ASD or ADHD | ASD sample: mean age 11.26 (3.63)  ADHD sample: mean age 9,87 (2,92) | 1133 with ASD and 1024 with ADHD, 121 with ADHD and ASD |
| 1. Leopold et al., 2019 | Longidutinal study of twin pairs assessed at 6 time points over 10 years | Colorado, USA | Disruptive Behavior Rating Scale (DBRS) completed by parents | Not specified | Community | Prior to starting kindergarten mean age 4.9 years (sd 0.2) | 489 twin pairs: 224 monozygtic and 265 dizygotic |
| 1. Lúcio et al., 2022 | Longitudinal: 2 repeated assessments | Brazil | Swanson, Noan and Pelahm scales version IV (SNAP-IV) for parents and teachers | 51.8% male | Community | Mean age 4.269 (sd 0.66) at the first time point | 618 |
| 1. MacDonald et al., 2019 | Longitudinal: 2 repeated assessments | Australia, Norway, Sweden and USA | Disruptive Bheavior Scale (DBRS) for parents na teahcers | Approximately 1:1 male-female ratio | Community twin sample | At initial contact, between the ages of 47 to 68 months. | 974 |
| 1. Makransky et al., 2014 | Cross-Sectional | Denmark | ADHD-RS for parents and teachers | 48% boys | Community | Mean age: 10.98 | 566 |
| 1. Narad et al., 2015 | Cross-Sectional | USA | Vanderbilt ADHD Rating Scales for parents (VADPRS) and teachers (VADTRS) | 68.2% male | Clinical | Mean age 9.34 (sd 3.03) | 6 659 |
| 1. Preszler et al., 2019 | Cross-Sectional | Brazil, USA and Thailand | Child and Adolescent Behavior Inventory (CADBI) for parents | 49.5% female | Community | Mean age: 9.04, sd 2.12 | 3785 |
| 1. Preszler et al., 2022 | Longitudinal study: 2 repeated assessments 6 weeks apart | Mallorca & Madrid, Spain | Child and Adolescent Behavior Inventory (CADBI) in parents | 46% female | Community | first-grade children | 802 |
| 1. Rodenacker et al., 2016 | Cross-Sectional | Germany | Fremdbeurteilungsbogen für Kinder and Jugendliche mit Aufmerksamkeitsdefizit-/Hyperaktivitätsstörung(FBB-ADHD) | Clinical: 76.5% male  Community sample: 48.8% males | Clinical and Community | Clinical: mean age 11.4, sd 3.1  Community: mean agre 11.2, sd 3.4 | Clinical (n=1 081  community sample (n=642 |
| 1. Toplak et al., 2012 | Cross-Sectional (IMAGE study) | Belgium, England Germany, Ireland, Spain, Switzerland, Netherlands and Israel | PACS Parent interview  Conners Parent Form  Conners Teacher Form | Clinical: 27% male  Unselected siblings: 50.2% male, | Clinical (probands) and unselected siblings | Clinical: mean age 10.95 (sd 2.78)  Unselected siblings: mean age: 10.87 (sd 3.36) | 1373 probands and 1772 unselected siblings |
| 1. Trejo et al., 2022 Dec 1. | Cross-Sectional | Mexico | Strengths and Weaknesses of ADHD-Symptoms and Normal Behavior Scale (SWAN) and Barrios and Matute Questionnaire of ADHD symptoms (BMQ-ADHD). | 55.84% female | Community | Mean age:13.15 (sd= 0.97) | 650 |
| 1. Vitoratou & Garcia‐Rosales et al., 2019 | Cross-Sectional, IMAGE study | Belgium, England Germany, Ireland, Spain, Switzerland, Netherlands and Israel | Hypescheme algorithm to ascertain symptom criteria as present or absent using Parental Account of Clinical Symptoms and the Conners Teacher Rating Scale: Revised-Long | 17.9% female | Clinical and siblings randomly selected with only one child per family | Mean age 10.9 sd 2.9 | 1 383 |
| 1. Willoughby et al., 2012 | Longitudinal study with 3 repeated yearly assessments. | 3 counties in Eastern North Carolina and 3 counties in Central Pennsylvania selected to be indicative of the Black South and Appalachia respectively. USA | ADHD Symptom Rating Scale Checklist | 50% female | Community | Initially: between ages 3-5 year old | 1155 |
