# Supplementary material for: Measurement invariance of Attention Deficit/Hyperactivity Disorder symptom criteria as rated by parents and teachers in children and adolescents: A systematic review
Source: PLoS One. 2024 Feb 23;19(2):e0293677. doi: 10.1371/journal.pone.0293677 (PMC10889893; doi:10.1371/journal.pone.0293677)
Supplement: S3 Table — (DOCX) [file pone.0293677.s006.docx]

| *Table S3 Summary table of the Age Invariance publications (total of 24 tests) with the number of comparisons depending on informant (parents or teachers).* | | | | | | |
| --- | --- | --- | --- | --- | --- | --- |
| **Publication** | **Sample** | **Model** | **Scale** | **Number of**  **comparisons** | **Parents** | **Teachers** |
| Burns et al., 1997 | Community and Clinical | 4-factor model; IA, HI, ODD and overt conduct disorder factors | Child and Adolescent Behavior Inventory (CADBI) for parents | 1 | 1 | 0 |
| Caci et al., 2016 | Community | 3-factor model with IA, HI and ODD | Child and Adolescent Disruptive Behavior Inventory (CADBI) parent version | 1 | 0 | 1 |
| Cogo-Moreira et al., 2019 | High-risk and random sample | Bi-factor model including one general ADHD facto and 3 specific factors (Inattentiveness, Hyperactivity and Impulsivity) | DAWBA administered to biological mother by trained lay interviewer | 1 | 1 | 0 |
| Dobrean et al., 2021 | Clinical and non-clinical groups | Two factor model with IA and HI | ADHD-RS-IV parent and teacher versions | 1 | 1 | 1 |
| DuPaul et al., 2016 | Community | Two factor model with IA and HI | ADHD Rating Scale-5 Home and School | 4 | 2 | 2 |
| DuPaul et al., 2020 | Community | Univariate for each subscale IA and HI. | ADHD Rating Scale-5 Home and School | 2 | 1 | 1 |
| Krakowski et al., 2022* | Clinical sample with diagnoses of ADHD or ASD | 4-factor model with social communication factor, restricted and repetitive interests factor the hyperactivity impulsivity factor and the inattentive factor. | Swanson, Nolan and Pelham scale version IV (SNAP-IV) for parents | 1 | 1 | 0 |
| Makransky & Bilenberg, 2014 | Community | Unidimensionalityf or each subscale IA, HI and ODD | ADHD-RS for parents and teachers | 2 | 1 | 1 |
| Rodenacker et al., 2016 | Clinical and community | Incomplete bifactor model | Fremdbeurteilungsbogen für Kinder and Jugendliche mit Aufmerksamkeitsdefizit-/Hyperaktivitätsstörung(FBB-ADHD) | 1 | 1 | 0 |
| Toplak et al., 2012 | Clinical sample with their siblings | Hierarchical model with 2 factors | PACS Parent interview, Conners for parents and teachers | 4 | 3 | 1 |
| Vitoratou & Garcia‐Rosales et al., 2020 | Clinical sample with their siblings | Two factor model with IA and HI | Hypescheme algorithm to ascertain symptom criteria as present or absent using Parental Account of Clinical Symptoms and the Conners Teacher | 2 | 1 | 1 |

* only includes 16 ADHD DSM-IV items
