# Supplementary material for: Measurement invariance of Attention Deficit/Hyperactivity Disorder symptom criteria as rated by parents and teachers in children and adolescents: A systematic review
Source: PLoS One. 2024 Feb 23;19(2):e0293677. doi: 10.1371/journal.pone.0293677 (PMC10889893; doi:10.1371/journal.pone.0293677)
Supplement: S4 Table — Where there is bias the direction of the bias is specified along the number of comparisons. Tables are elaborated based on the available and reported data. (DOCX) [file pone.0293677.s007.docx]

| *Table S4 Measurement (Non)-Invariance assessment: Younger (Y; less than 10 years old) versus Older (O; 11 years old and older) according to Parents. Where there is bias the direction of the bias is specified along the number of comparisons. Tables are elaborated based on the available and reported data.* | | | | | | |
| --- | --- | --- | --- | --- | --- | --- |
| **Symptom criterion** | **Metric (weak) invariance** | | | **Scalar (strong) invariance** | | |
|  | ***Number of***  ***Comparisons*** | ***Invariant loadings*** | ***Direction of bias*** | ***Number of***  ***Comparisons*** | ***Invariant thresholds*** | ***Direction of bias*** |
| **Inattentiveness** | | | | | | |
| *Careless* | 12 | 12 |  | 12 | 11 | ^1^O>Y: 1 |
| *Attention* | 13 | 13 |  | 12 | 11 | Y>O: 1 |
| *Listens* | 11 | 11 |  | 10 | 9 | ^1^Y>O: 1 |
| *Instructions* | 13 | 13 |  | 12 | 11 | Y >O: 1 |
| *Disorganised* | 13 | 13 |  | 12 | 12 |  |
| *Unmotivated* | 12 | 12 |  | 12 | 10 | Y >O: 1; O>Y: 1 |
| *Loses* | 13 | 12 | Y>O: 1 | 12 | 12 |  |
| *Distracted* | 13 | 13 |  | 12 | 11 | Y >O: 1 |
| *Forgetful* | 12 | 12 |  | 12 | 12 |  |
| **Hyperactivity/Impulsivity** | | | | | | |
| *Fidgets* | 13 | 13 |  | 11 | 10 | O>Y: 1 |
| *Seats* | 12 | 12 |  | 11 | 10 | Y>O: 1 |
| *Runs/Climbs* | 12 | 12 |  | 12 | 10 | ^2^Y>O: 2 |
| *Quiet* | 13 | 13 |  | 12 | 12 |  |
| *Motor* | 12 | 12 |  | 12 | 11 | ^2^O>Y: 1 |
| *Talks* | 13 | 13 |  | 12 | 12 |  |
| *Blurts* | 12 | 12 |  | 11 | 10 | ^2^O>Y: 1 |
| *Wait* | 13 | 13 |  | 12 | 12 |  |
| *Interrupts* | 11 | 11 |  | 11 | 11 |  |
