# Supplementary material for: Measurement invariance of Attention Deficit/Hyperactivity Disorder symptom criteria as rated by parents and teachers in children and adolescents: A systematic review
Source: PLoS One. 2024 Feb 23;19(2):e0293677. doi: 10.1371/journal.pone.0293677 (PMC10889893; doi:10.1371/journal.pone.0293677)
Supplement: S5 Table — Where there is bias the direction of the bias is specified along the number of comparisons. (DOCX) [file pone.0293677.s008.docx]

| *Table S5 Measurement (Non)-Invariance assessment: Younger (Y; less than 10 years old) versus Older (O; 11 years old and older) according to Teachers. Where there is bias the direction of the bias is specified along the number of comparisons.* | | | | | | |
| --- | --- | --- | --- | --- | --- | --- |
| **Symptom criterion** | **Metric (weak) invariance** | | | **Scalar (strong) invariance** | | |
|  | ***Number of***  ***Comparisons*** | ***Invariant loadings*** | ***Direction of bias*** | ***Number of***  ***Comparisons*** | ***Invariant thresholds*** | ***Direction of bias*** |
| **Inattentiveness** | | | | | | |
| Careless | 8 | 8 |  | 8 | 8 |  |
| Attention | 8 | 8 |  | 8 | 8 |  |
| Listens | 8 | 8 |  | 8 | 8 |  |
| Instructions | 8 | 8 |  | 8 | 7 | ^1^Y >O: 1 |
| Disorganised | 8 | 8 |  | 8 | 8 |  |
| Unmotivated | 8 | 8 |  | 8 | 8 |  |
| Loses | 8 | 8 |  | 8 | 7 | ^1^O>Y: 1 |
| Distracted | 8 | 8 |  | 8 | 6 | ^1^Y>O: 2 |
| Forgetful | 8 | 8 |  | 7 | 6 | ^2^O>Y: 1 |
| **Hyperactivity/Impulsivity** | | | | | | |
| Fidgets | 8 | 8 |  | 8 | 8 |  |
| Seats | 8 | 8 |  | 8 | 8 |  |
| Runs/Climbs | 8 | 8 |  | 8 | 8 |  |
| Quiet | 8 | 8 |  | 8 | 8 |  |
| Motor | 8 | 8 |  | 8 | 8 |  |
| Talks | 8 | 8 |  | 8 | 7 | ^2^O>Y: 1 |
| Blurts | 8 | 8 |  | 8 | 7 | ^2^O>Y: 1 |
| Wait | 8 | 8 |  | 8 | 7 | ^2^O>Y: 1 |
| Interrupts | 8 | 8 |  | 8 | 7 | ^2^O>Y: 1 |
| ^1^DIF ^2^MIMIC | | | | | | |
