# Supplementary material for: Measurement invariance of Attention Deficit/Hyperactivity Disorder symptom criteria as rated by parents and teachers in children and adolescents: A systematic review
Source: PLoS One. 2024 Feb 23;19(2):e0293677. doi: 10.1371/journal.pone.0293677 (PMC10889893; doi:10.1371/journal.pone.0293677)
Supplement: S6 Table — (DOCX) [file pone.0293677.s009.docx]

| *Table S6 Summary table of the Temporal (longitudinal) Invariance publications (total of 14 tests) with the number of comparisons depending on informant (parents or teachers).* | | | | | | | | | |
| --- | --- | --- | --- | --- | --- | --- | --- | --- | --- |
| **Publication** | **Sample** | **Model** | **Scale** | **Number of**  **comparisons** | **Parents** | **Teachers** | **Combined parents and teachers** | **Mothers**  **only** | **Fathers only** |
| Gomez et al., 2018 | Clinic-referred sample | Bifactor model for IA and HI | Strengths and Weaknesses of ADHD-Symptoms and Normal Behavior Scale (SWAN) in mothers | 1 | 1 |  |  |  |  |
| Hall et al., 2020 | Clinical | 2-factor model with IA and HI | Swanson, Nolan and Pelham Rating Scale (SNAP-IV) for parents and teachers | 4 | 1 | 2 | 1 |  |  |
| Leopold et al., 2019 | Twin pairs | 3 factor model with IN, HI and Functional Impairment | Disruptive BehaviorRating Scale (DBRS) completed by parents | 1 | 1 |  |  |  |  |
| Lúcio et al., 2022 | Community | 3 correlated factors solution with IA, HI and ODD | Swanson, Nolan and Pelham scale version IV (SNAP-IV) for parents and teachers | 2 | 2 | 2 |  |  |  |
| MacDonald et al., 2019 | Twins | 2-factor model with IA and HI | Disruptive Behavior Scale (DBRS) for parents and teachers | 3 | 3 |  |  |  |  |
| Preszler et al., 2022. | School sample | 3 factor model with cross-loadings including IA, HI and Impulsivity | Child and Adolescent Behavior Inventory (CADBI) for parents | 2 |  |  |  | 1 | 1 |
| Willoughby et al., 2012 | Community | 1-factor model | ADHD Symptom Rating Scale Checklist | 1 | 1 |  |  |  |  |
