# Supplementary material for: Measurement invariance of Attention Deficit/Hyperactivity Disorder symptom criteria as rated by parents and teachers in children and adolescents: A systematic review
Source: PLoS One. 2024 Feb 23;19(2):e0293677. doi: 10.1371/journal.pone.0293677 (PMC10889893; doi:10.1371/journal.pone.0293677)
Supplement: S7 Table — Where there is bias the direction of the bias is specified along the number of comparisons. (DOCX) [file pone.0293677.s010.docx]

| *Table S7: Measurement (Non)-Invariance assessment: Repeated assessments according to Parents. Where there is bias the direction of the bias is specified along the number of comparisons.* | | | | | | | | | | | | |  |
| --- | --- | --- | --- | --- | --- | --- | --- | --- | --- | --- | --- | --- | --- |
| **Symptom criterion** | **Metric (weak) invariance** | | | | | | **Scalar (strong) invariance** | | | | | |  |
|  | ***Number of***  ***Comparisons*** | | ***Invariant loadings*** | | ***Direction of bias*** | | ***Number of***  ***Comparisons*** | | ***Invariant thresholds*** | | ***Direction of bias*** | |  |
| **Inattentiveness** | | | | | | | | | | | | |  |
| *Careless* | 7 | | 6 | |  | | 6 | | 3 | |  | |  |
| *Attention* | 7 | | 6 | |  | | 6 | | 3 | |  | |  |
| *Listens* | 7 | | 6 | |  | | 6 | | 3 | |  | |  |
| *Instructions* | 7 | | 6 | |  | | 6 | | 3 | |  | |  |
| *Disorganised* | 7 | | 6 | |  | | 6 | | 3 | |  | |  |
| *Unmotivated* | 7 | | 6 | |  | | 6 | | 3 | |  | |  |
| *Loses* | 7 | | 6 | |  | | 6 | | 3 | |  | |  |
| *Distracted* | 7 | | 6 | |  | | 6 | | 3 | |  | |  |
| *Forgetful* | 7 | | 5 | |  | | 5 | | 3 | |  | |  |
| **Hyperactivity/Impulsivity** | | | | | | | | | | | | |  |
| *Fidgets* | 7 | | 5 | |  | | 5 | | 3 | |  | |  |
| *Seats* | 7 | | 5 | |  | | 5 | | 3 | |  | |  |
| *Runs/Climbs* | 7 | | 5 | |  | | 5 | | 3 | |  | |  |
| *Quiet* | 7 | | 5 | |  | | 5 | | 3 | |  | |  |
| *Motor* | 7 | | 4 | |  | | 4 | | 4 | |  | |  |
| *Talks* | 7 | | 5 | |  | | 6 | | 3 | |  | |  |
| *Blurts* | 7 | | 5 | |  | | 5 | | 3 | |  | |  |
| *Wait* | 7 | | 5 | |  | | 5 | | 3 | |  | |  |
| *Interrupts* | 7 | | 5 | |  | | 5 | | 3 | |  | |  |
| *Motor* | | 4 | | 3 | |  | | 3 | | 3 | |  | |
| *Talks* | | 4 | | 3 | |  | | 3 | | 3 | |  | |
| *Blurts* | | 4 | | 3 | |  | | 3 | | 3 | |  | |
| *Wait* | | 4 | | 3 | |  | | 3 | | 3 | |  | |
| *Interrupts* | | 4 | | 3 | |  | | 3 | | 3 | |  | |
