# Supplementary material for: Measurement invariance of Attention Deficit/Hyperactivity Disorder symptom criteria as rated by parents and teachers in children and adolescents: A systematic review
Source: PLoS One. 2024 Feb 23;19(2):e0293677. doi: 10.1371/journal.pone.0293677 (PMC10889893; doi:10.1371/journal.pone.0293677)
Supplement: S8 Table — Where there is bias the direction of the bias is specified along the number of comparisons. (DOCX) [file pone.0293677.s011.docx]

| *Table S8 Measurement (Non)-Invariance assessment: Repeated assessments according to Teachers. Where there is bias the direction of the bias is specified along the number of comparisons.* | | | | | | |
| --- | --- | --- | --- | --- | --- | --- |
| **Symptom criterion** | **Metric (weak) invariance** | | | **Scalar (strong) invariance** | | |
|  | ***Number of***  ***Comparisons*** | ***Invariant loadings*** | ***Direction of bias*** | ***Number of***  ***Comparisons*** | ***Invariant thresholds*** | ***Direction of bias*** |
| **Inattentiveness** | | | | | | |
| *Careless* | 4 | 3 |  | 3 | 3 |  |
| *Attention* | 4 | 3 |  | 3 | 3 |  |
| *Listens* | 4 | 3 |  | 3 | 3 |  |
| *Instructions* | 4 | 3 |  | 3 | 3 |  |
| *Disorganised* | 4 | 3 |  | 3 | 3 |  |
| *Unmotivated* | 4 | 3 |  | 3 | 3 |  |
| *Loses* | 4 | 3 |  | 3 | 3 |  |
| *Distracted* | 4 | 3 |  | 3 | 3 |  |
| *Forgetful* | 4 | 3 |  | 3 | 3 |  |
| **Hyperactivity/Impulsivity** | | | | | | |
| *Fidgets* | 4 | 3 |  | 3 | 3 |  |
| *Seats* | 4 | 3 |  | 3 | 3 |  |
| *Runs/Climbs* | 4 | 3 |  | 3 | 3 |  |
| *Quiet* | 4 | 3 |  | 3 | 3 |  |
| *Motor* | 4 | 3 |  | 3 | 3 |  |
| *Talks* | 4 | 3 |  | 3 | 3 |  |
| *Blurts* | 4 | 3 |  | 3 | 3 |  |
| *Wait* | 4 | 3 |  | 3 | 3 |  |
| *Interrupts* | 4 | 3 |  | 3 | 3 |  |
